# Prognostic significance and immune infiltration analysis of HMGA2 in endometrial cancer

**DOI:** 10.3389/fimmu.2025.1559278

**Published:** 2025-07-09

**Authors:** Peng Jiang, Jiaxin Yu, Yunfeng Zheng, Chenfan Tian, Yuan Tu, Chunxia Gong, Hangkun Yu, Yi Luo, Zhuoying Hu

**Affiliations:** ^1^ Department of Gynecology, The First Affiliated Hospital of Chongqing Medical University, Chongqing, China; ^2^ Department of Gynecology, Women and Children’s Hospital of Chongqing Medical University, Chongqing, China

**Keywords:** endometrial cancer, HMGA2, immune, prognostic value, macropha ge polarization

## Abstract

**Background:**

HMGA2, as a transcription factor, facilitates oncogenesis and malignant progression by coordinating cell cycle dysregulation, compromising DNA repair machinery, and suppressing cancer cell apoptosis. However, its roles in prognostication and tumor immune microenvironment modulation in endometrial cancer (EC) remain incompletely defined.

**Methods:**

We systematically analyzed HMGA2 expression patterns and clinical prognostic value in EC using bioinformatics strategies, including TCGA and GTEX data mining, as well as single gene expression analysis. Functional enrichment analysis (GSEA and KEGG) identified HMGA2-associated pathways. The correlation between HMGA2 and immune infiltration was assessed via TIMER and TISIDB. Subsequent *in vitro* (proliferation, migration, colony formation) and *in vivo* (xenograft models) experimental were used to validate HMGA2’s role in promoting EC progression. The correlation between HMGA2 and macrophage markers (CD86 and CD206) was validated through clinical tissue samples by IHC. Finally, a recurrence-predictive nomogram incorporating HMGA2 with clinicopathological parameters was established.

**Results:**

HMGA2 exhibited significant upregulation in endometrial cancer (EC) tissues and correlated with poor patient prognosis. Immunoassay showed that high expression of HMGA2 was negatively correlated with infiltration of various immune cells, especially M1 macrophages. Cytological experiments showed that knocking down HMGA2 significantly inhibited EC cell proliferation, migration, invasion, and drug resistance, while overexpression of HMGA2 promoted the above phenotype; Animal experiments showed that knocking down HMGA2 significantly inhibited the growth of EC tumors and the expression of M1 macrophage marker CD86. The combination of HMGA2 inhibitors and targeted macrophage immunotherapy (CD47 monoclonal antibody) had the better tumor suppression effect. Clinical sample analysis found that high expression of HMGA2 was significantly negatively correlated with CD86 and positively correlated with CD206 expression. Patients with low HMGA2 expression showed enhanced immune therapy responsiveness. The nomogram model based on HMGA2 and clinical pathological parameters showed better predictive performance (AUC=0.855, sensitivity=79.0%, specificity=76.8%).

**Conclusion:**

HMGA2 is a potential diagnostic and prognostic biomarker for the EC. HMGA2 may drive the occurrence and development of EC by inhibiting the infiltration of immune cells, especially M1 macrophages. Therapeutic targeting of HMGA2 is a novel strategy for EC intervention.

## 1 Introduction

Endometrial cancer (EC) ranks among the most prevalent gynecologic malignancies. Over the past three decades, its global incidence has surged by 132%, with a growing prevalence among younger women - particularly notable in patients under 40 years where case numbers have doubled (1). While two-thirds of EC patients present with early-stage disease amenable to surgical cure, those with advanced or recurrent disease face poor outcomes. Current management of metastatic EC increasingly incorporates targeted therapies against VEGF, mTOR, and immune checkpoints (2, 3). Despite therapeutic advances, the identification of novel biomarkers remains crucial for improving EC diagnosis and personalized treatment strategies.

HMGA2, a chromatin-remodeling protein encoded on chromosome 12q13–15, regulates transcription via AT-hook-mediated DNA binding (4, 5). Crucially, this oncoprotein exhibits near-negligible expression in normal tissues but is aberrantly overexpressed across multiple malignancies—including breast, ovarian, and lung cancers—where it drives tumorigenesis through cell-cycle dysregulation, apoptosis suppression, and DNA repair alteration (4, 6–8). These findings establish HMGA2 as a multifunctional oncogenic driver and emerging therapeutic target in solid tumors.

Despite its documented roles in other cancers, HMGA2’s prognostic and immunomodulatory functions in EC remain poorly defined. Preliminary evidence suggested that the overexpression of HMGA2, through the control of transcription, is related to the pathogenesis of EC (9). Given EC’s rising incidence and the urgent need for biomarkers guiding immunotherapy deployment, systematic characterization of HMGA2 represents a critical research priority for optimizing risk-adapted therapy.

This study investigates HMGA2’s prognostic value and immunomodulatory roles in EC. Our preliminary data suggested HMGA2 contributed to the occurrence and development of EC through transcriptional regulation of tumor proliferation and immune microenvironment suppression. We further developed an integrated prognostic model combining HMGA2 expression with clinicopathological features to identify high-risk patients for tailored clinical management.

## 2 Materials and methods

### 2.1 Bioinformatics tools for analyzing the differential expression of HMGA2

Differential expression of HMGA2 between endometrial carcinomas and adjacent normal tissues was initially analyzed using the Diff Exp module of TIMER. Pan-cancer validation was subsequently performed via the cross-cancer analysis module of Sangerbox. For TCGA-UCEC cohort-specific profiling, UALCAN (a web-based platform for multi-omics TCGA data mining) was employed to quantify HMGA2 expression levels across FIGO stages and histological subtypes, simultaneously assessing its promoter methylation status. Associations with molecular subtypes were investigated using TISIDB. Protein-level validation was conducted through immunohistochemical images of clinical specimens retrieved from The Human Protein Atlas (HPA) (10), which integrates transcriptomic and proteomic data for spatial tissue mapping. For specific websites information for all bioinformatics tools was provided in **Supplementary Table 1**.

### 2.2 Kaplan–Meier survival analysis

Kaplan-Meier Plotter (11) was used to analyze the overall survival (OS) and recurrence-free survival (RFS) of HMGA2 in endometrial cancer to evaluate the prognostic of HMGA2 (patients were divided into HMGA2-high/low groups by optimal cut-off values).

### 2.3 PPI network and functional enrichment analysis

The protein-protein interaction (PPI) network of HMGA2 was generated using GeneMANIA (12) with the following parameters: physical interactions, co-expression, and genetic interactions sourced from BioGRID, IMEx, and GEO datasets; an algorithm-optimized weighting (FDR<0.05 implicit filter); and a maximum resultant gene limit of 20. HMGA2 was specified as the primary query node. Subsequently, HMGA2-coexpressed genes were retrieved from cBioPortal (13), selecting the top 1,000 genes ranked by statistical significance (Spearman’s correlation; *P*<0.05). Following co-expression network construction, these genes were subjected to functional enrichment analysis via Metascape (14), performing Gene Ontology (GO) annotation and Kyoto Encyclopedia of Genes and Genomes (KEGG) pathway analysis. Results were visualized using Wei Sheng Xin (15), a cloud-based bioinformatics platform for analytical graphics.

### 2.4 Immune infiltration analysis

To delineate the relationship between HMGA2 and tumor immune microenvironment in endometrial carcinoma, transcriptomic data from TCGA-UCEC (via TIMER) were analyzed. Immune infiltration metrics, including immune/stromal/ESTIMATE scores, were quantified using the ESTIMATE algorithm (16). Tumor purity was computationally derived as: *Purity = cos (0.6049872018 + 0.000146788 × ESTIMATE score)* (17). The differential expression of immune/stromal/ESTIMATE scores and tumor purity between high and low HMGA2 groups were evaluated, and the survival differences in patients with different immune scores were explored. TISIDB (18) integrates multiple databases including TCGA and PubMed, allowing for the pre-calculation of the correlation between genes and immune functions for various tumors. We evaluated HMGA2 associations with tumor-infiltrating lymphocytes abundance, immune checkpoint inhibitors, and immunostimulatory molecules.

### 2.5 Immunotherapy response and chemotherapy response

Utilizing the Genomics of Drug Sensitivity in Cancer (GDSC) database (19), which systematically catalogs tumor cell drug response profiles for therapeutic target discovery, we evaluated correlations between HMGA2 expression and pharmacodynamic indicators. Specifically, Spearman analyses determined relationships with: (i) chemotherapeutic sensitivity (paclitaxel and cisplatin) *in vitro* efficacy, quantified by half-maximal inhibitory concentration [IC50], where lower IC50 values denote enhanced sensitivity); and (ii) immune checkpoint inhibitor responsiveness (predicted via Tumor Immune Dysfunction and Exclusion [TIDE] scores, wherein higher scores indicate poorer immunotherapy sensitivity).

### 2.6 Gene set enrichment analysis

Gene set enrichment analysis (GSEA) (20) was performed using the C5 and BioCarta gene sets in GSEA software (version 4.3.2), with a statistical significance threshold of P<0.05.

### 2.7 Patient cohort

This retrospective cohort study enrolled stage I-III endometrial carcinoma patients (staged per 2009 FIGO criteria (21) who underwent primary surgical resection between January 2018 and December 2021 at the First Affiliated Hospital and Women and Children’s Hospital of Chongqing Medical University. Exclusion criteria comprised: non-standard surgical management; sarcomatous histopathology; metastatic disease; synchronous malignancies; neoadjuvant chemo/radiotherapy; or loss to follow-up. For all included cases, clinical records were collected, encompassing demographic parameters (age, BMI), pathological characteristics (histological subtype/grade, myometrial/cervical stromal invasion, lymphovascular space invasion [LVSI]), serum CA125 levels, and adjuvant therapy regimen after surgery.

Prepare paraffin sections of postoperative tissue specimens from patients stored in the pathology center for subsequent immunohistochemical analysis. Additionally, 30 pairs of fresh cancer tissues and adjacent normal endometrial tissues were collected for subsequent qRT-PCR and Western Blotting experiments. The postoperative adjuvant therapy, follow-up, and definition of recurrence for patients can be found in previously published literature (22). This study was conducted in compliance with the Declaration of Helsinki and obtained ethics clearance (Approval No. 2024-315–01 and 2023-002) from the institutional review boards of all participating hospitals.

### 2.8 Immunohistochemistry

Patient pathological paraffin sections were obtained from the pathology center for IHC analysis, following the same steps as previously published literature (23). The following primary antibodies are used for IHC staining: HMGA2 (Abcam, ab97276; 1:500 dilution), Ki-67 (Proteintech, 27309-1-AP; 1:1000 dilution), CD86 (Abcam, ab243887; 1:500 dilution), and CD206 (Proteintech, 18704-1-AP; 1:4000 dilution). Immunohistochemical assessments were performed according to standardized protocols. For HMGA2 and Ki-67, tumor cells exhibiting nuclear brownish-yellow granular staining were defined as positive. Five randomly selected high-power fields (40× objective) were evaluated in maximally active tumor regions. Per field, 100 tumor cells were counted to calculate the mean positive percentage (0-100%). Staining intensity was stratified into four-tiered scoring: 1 (negative), 2 (weak), 3 (positive), and 4 (strong). The final histoscore (range 1-4) was computed as: [staining intensity score] × [positive percentage]. P53 expression was classified as mutant-type when demonstrating complete absence or strong diffuse nuclear staining (>80% cells), otherwise wild-type. CD86^+^ and CD206^+^ cells were manually quantified across entire 1 mm cores with results expressed as mean counts per mm² (24). All sections underwent blind independent evaluation by two certified pathologists, with discordances resolved through joint re-examination (25).

### 2.9 Cell culture

Human endometrial cancer cell lines Ishikawa and HEC1-A were purchased from Nanjing Baso Biotechnology Co., Ltd. (Nanjing, China). Cells were cultured using DMEM medium (Gibco, China) containing 10% fetal bovine serum (FBS) (UElandy, Suzhou, China) and 2% penicillin-streptomycin (Biosharp, Beijing, China), and placed in a humidified incubator (Thermo Scientific, China) at 37°C with 5% CO_2_ (26).

### 2.10 Transfection

HMGA2 lentivirus was Obtained from Gikai Biotechnology Company. Ishikawa and HEC1-A cells were inoculated in a six-well plate and culture overnight to achieve approximately 30% confluence after 24 hours, then the lentivirus was transduced according to the manufacturer’s instructions (MOI=20). The culture medium was changed after 24 hours of transfection, and then continued to expand the cells. A portion of the cells were taken for qRT-PCR and WB to verify transfection efficiency (27). The sequence of HMGA2 siRNA: shHMGA2#1, AGTCCCTCTAAAGCAGCTCAA; shHMGA2#2, CTCCTAAGAGACCCAGGGGAA.

### 2.11 Western blotting

Following cell lysis, supernatants were centrifuged (12,000 ×g, 10 min, 4°C) and protein concentrations determined using a bicinchoninic acid (BCA) assay kit (Beyotime Biotechnology, China). Protein lysates (30 μg per lane) were resolved on 10% SDS-PAGE gels at 80 V for 30 min followed by 120 V for 90 min, then electrotransferred onto PVDF membranes (250 mA, 80 min). Membranes were blocked in 5% (w/v) non-fat dry milk/TBST for 1.5 h at 25°C prior to overnight incubation at 4°C with primary antibodies prepared in blocking buffer: anti-HMGA2 (Abcam, ab97276; 1:10,000) anti-Ki-67 (Santa Cruz, sc-23900; 1:500) anti-N-cadherin (Proteintech, 82968-1-RR; 1:10000) anti-E-cadherin (Proteintech, 20874-1-AP; 1:50000) anti-Vimentin (Proteintech, 80232-1-RR; 1:20000) and anti-GAPDH (Proteintech, 60004-1-Ig; 1:50000). After TBST washes (3 × 10 min), membranes were probed with HRP-conjugated secondary antibodies (goat anti-mouse IgG, ZenBio ASG031N, 1:10000 or goat anti-rabbit IgG, ZenBio N19JU46, 1:50000) at 25°C for 60 min. Protein bands were detected using ECL substrate with chemiluminescent imaging performed on an Azure c600 system (28).

### 2.12 RNA extraction and quantitative real-time PCR

Total RNA was extracted from cell lines using trizol reagent (vazyme, China). ABScript III RT Master Mix (MCE, USA) with gDNA remover was performed for qPCR, cDNA was synthesized with random primers; qRT-PCR reactions were performed with BrightCycle Universal SYBR Green qPCR Mix with UDG (MCE, USA). β-actin was used as an internal control. The primers were synthesized by Chengdu Meiji (28). Primer sequences were as follows: HMGA2: Sense primer: 5’-CGGTGAGCCCTCTCCTAAG-3’; Anti-sense primer: 5’-CTCCAGTGGCTTCTGCTTTC-3’; β-actin: Sense primer: 5’-AGAAAATCTGGCACCACACCT-3’; Anti-sense primer: 5’-GATAGCACAGCCTGGATAGCA-3’.

### 2.13 Proliferation, migration and invasion assays

Cellular proliferation was assessed using a CCK-8 assay (Dogindo, China). Briefly, 1.0 × 10³ cells/well were seeded in 96-well plates and pre-cultured for 24 h at 37°C under 5% CO_2_. At 0, 24, 48, and 72 h post-seeding, cells were incubated with 100 µl of CCK-8 reagent diluted 1:10 in serum-free medium for 2 h at 37°C. Absorbance was measured at 450 nm using a microplate reader (Thermo Scientific, China), with blank subtraction for normalization.

Migration capacity was evaluated via scratch assay. Cells grew in 6-well plates until ≥90% confluency. A standardized wound was created using a sterile 200 µl pipette tip (mean scratch width: 500 ± 50 µm). After washing with PBS, cells were maintained in serum-free medium. Migratory progression was documented at 0 and 48 h using an inverted phase-contrast microscope (Olympus IX73). Quantification was performed by calculating wound closure rate: % Closure = [(A_0_ − A_48_)/A_0_] × 100, where A represents wound area measured with ImageJ software.

Invasion potential was analyzed using Matrigel-coated Transwell chambers (Corning, 8 μm pores). Cells (1 × 10^4^/well) in serum-free DMEM were plated in the upper chamber, with DMEM containing 30% FBS as chemoattractant in the lower compartment. After 24 h incubation at 37°C/5% CO_2_, invaded cells were fixed with 4% paraformaldehyde (Biosharp, China) for 15 min, stained with 0.1% crystal violet (Beyotime, China) for 20 min, and washed three times with PBS. Cell counts were obtained from ≥3 random fields per chamber under an inverted microscope at 100× magnification (29).

### 2.14 Colony formation assay

Ishikawa and HEC1-A cells were seeded in 6-well plates at 3,000 cells/well. Following 24-hour adherence, cells were treated with paclitaxel (HY-B0015, MedChemExpress), cisplatin (HY-17394, MedChemExpress), and ciclopirox (CPX, an inhibitor targeting HMGA2) (HY-B0450, MedChemExpress) at specified concentrations for 14 days (30–32). Colonies were then fixed with 4% paraformaldehyde (White Shark, China) for 20 min, stained with 0.1% crystal violet (Beyotime, China) for 30 min, and quantified by counting macroscopic colonies containing >50 cells (diameter ≥0.5 mm). Three independent biological replicates were performed, with colony counts expressed as mean ± standard deviation (33).

### 2.15 Subcutaneous tumor model of xenotransplantation

Subcutaneous xenograft models were established in 4-week-old female BALB/c nude mice (n=5/group; Chengdu Yaokang Biotechnology, China) by injecting 1×10^7^ lentivirus-transduced EC stable cells suspended in 100 μL PBS into the right thigh region. Tumor progression was monitored twice weekly via digital caliper measurements, with volumes calculated as 0.5 × (major axis) × (minor axis)^2^. Mice were humanely euthanized by cervical dislocation under isoflurane anesthesia after 4 weeks.

Fifteen 4-week-old female BALB/c nude mice underwent subcutaneous inoculation of Ishikawa cells into the right thigh. Seven days post-implantation, mice were randomized into three experimental groups (n=5 per group): (1) vehicle control; (2) CPX (HY-B0450, MedChemExpress, 100 mg/kg oral gavage three times daily); and (3) CPX (100 mg/kg oral gavage t.i.d.) + intraperitoneal anti-human CD47 mAb (HY-P99706, MCE, 1 mg/kg twice weekly) (32, 34). Following 21 days of intervention, euthanasia was performed prior to tumor excision, with subsequent volumetric analysis via caliper measurement using the formula 0.5 × maximal diameter × (perpendicular diameter) ². All animal experimental procedures were approved by the Animal Ethics Committee of Chongqing Medical University (Approval No. IACUC-CQMU-2024-0991) and were performed in accordance with the institution’s guidelines for the care and use of laboratory animals.

### 2.16 Establishment and validation of the nomogram model

The training cohort (n=560) comprising endometrial cancer (EC) patients from the First Affiliated Hospital of Chongqing Medical University was utilized to develop and internally validate a recurrence prediction nomogram. External validation employed an independent cohort (n=272) from the affiliated Women and Children’s Hospital. Univariate Cox regression initially screened HMGA2 expression and clinicopathological parameters for recurrence association (P<0.05 retention threshold). Significant predictors subsequently entered multivariate Cox analysis to identify independent factors (final inclusion criterion: P<0.05). These predictors were integrated into a nomogram constructed with R 4.3.2 (survival and rms packages). The optimal 3-year recurrence-free survival (RFS) risk threshold was determined by maximizing the Youden index via ROC analysis. According to this threshold, both cohorts were stratified into high- and low-risk groups. Kaplan-Meier curves with log-rank tests compared RFS distributions, while calibration plots assessed concordance between nomogram-predicted probabilities and actuarial recurrence rates.

### 2.17 Statistical analysis

To compare the differences between the two groups, the chi-square test was used for categorical variables, and the t-test and rank-sum test were used for continuous variables. Statistical analysis was conducted using the SPSS Statistics 27.0 (IBM statistics, Chicago, Illinois, USA) and the GraphPad Prism 9.0 software (La Jolla, CA, USA). All experiments were performed in triplicate. P-value <0.05 was considered to indicate a statistically significant difference.

## 3 Results

### 3.1 Differential expression and prognostic analysis of HMGA2 in EC

**Figure 1:**
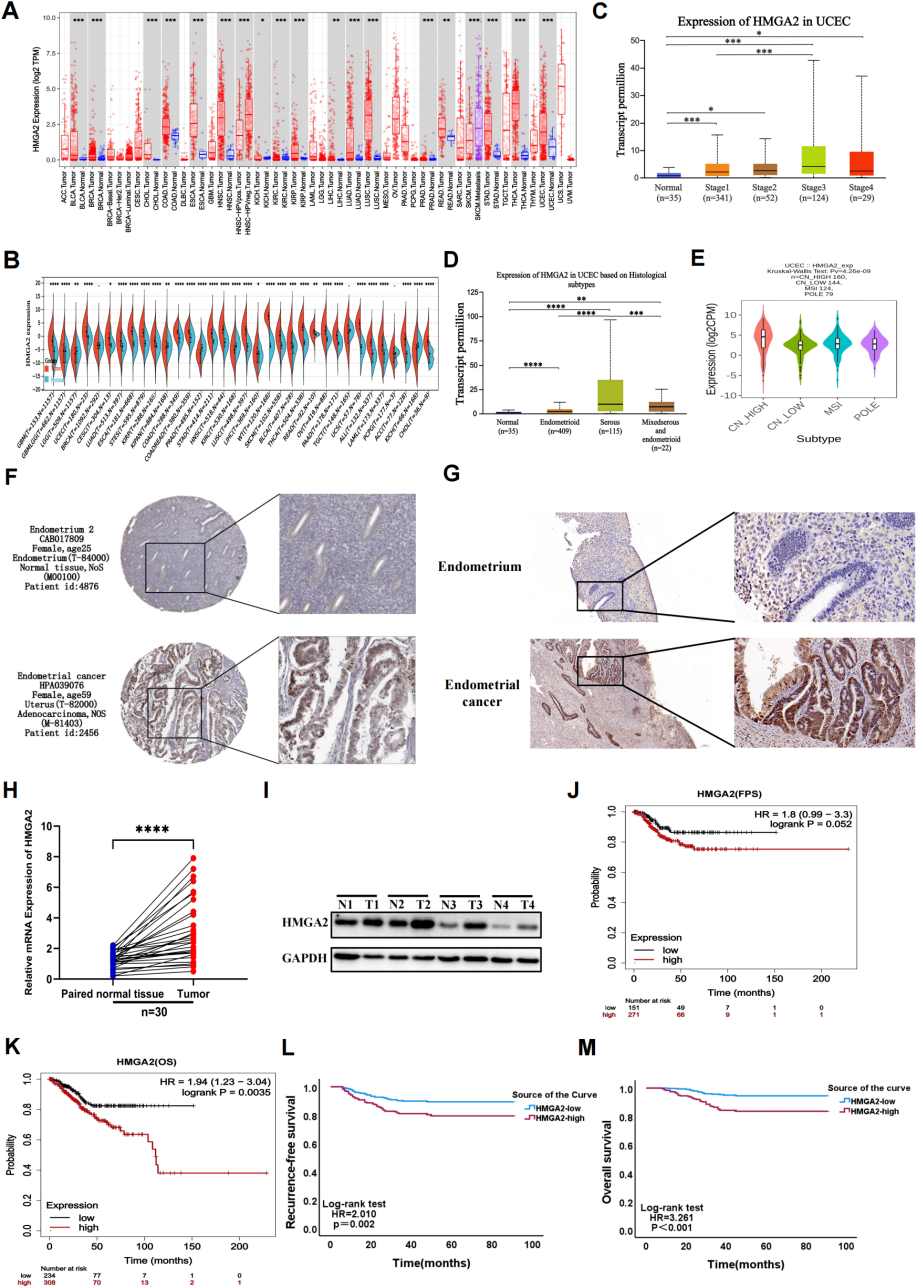
Gene expression and prognostic value of HMGA2 in UCEC patients. **(A, B)** Pan-cancer analysis of HMGA2 expression across RNA-seq datasets from TCGA. **(C)** Expression of HMGA2 in normal endometrial tissue and in different stages of endometrial cancer. **(D)** Expression of HMGA2 in normal endometrial tissue and different pathological types of endometrial cancer. **(E)** Expression of HMGA2 in different molecular subtypes of endometrial cancer. **(F, G)** Immunohistochemistry of HMGA2 expression in normal endometrial tissues and UCEC tissues based on HPA database and 30 pairs of tumor and adjacent tissues from our hospital. **(H, I)** The expression of HMGA2 in 30 pairs of UCEC tissues compared with adjacent tissues via RT-qPCR and WB. **(J-M**) Kaplan–Meier analysis of HMGA2 expression with recurrence-free survival (RFS) and overall survival (OS) values in UCEC patients based on TCGA and real data from our hospital. Notes: (*p < 0.05, **p < 0.01, ***p < 0.001, ****p < 0.0001, ns, not significant).

Comprehensive pan-cancer analysis of TCGA data revealed significant HMGA2 upregulation in multiple malignancies compared to matched normal tissues, including endometrial cancer (**Figures 1A, B**). HMGA2 expression demonstrated strong correlations with advanced tumor staging, aggressive molecular subtypes, and high-grade histopathology (**Figures 1C–E**). Immunohistochemical validation using HPA samples showed weak cytoplasmic staining in normal endometrium, contrasting with moderate-to-strong immunoreactivity in UCEC specimens (**Figure 1F**). This differential expression pattern was consistently replicated in our institutional cohort (**Figure 1G**), molecular confirmation via RT-qPCR and western blotting of 30 matched tumor-normal pairs confirmed profound HMGA2 transcript and protein overexpression in EC tissues (**Figures 1H, I**).

Importantly, HMGA2 overexpression in TCGA-UCEC dataset correlated with key clinicopathological risk factors including histological subtypes (P=0.029) and grade (P<0.001), TP53 mutation status (P<0.001), and adjuvant radiotherapy requirement (P=0.030; **Supplementary Table 2**). These associations were externally validated in our institutional cohort, where high HMGA2 expression consistently correlated with aggressive disease features including FIGO stage (P<0.001), LVSI (P=0.004), histological subtypes (P=0.048) and adjuvant treatment (P=0.002, **Supplementary Table 3**). Kaplan-Meier analyses further established HMGA2 as a robust prognostic determinant, wherein high expressers exhibited significantly reduced RFS (TCGA: HR=1.8, P=0.052; institutional cohort: HR=2.010, P=0.002) and OS (TCGA: HR=1.94, P=0.0035; institutional cohort: HR=3.261, P<0.001), with concordant findings across both cohorts (**Figures 1J–M**).

### 3.2 DNA methylation and function enrichment of HMGA2 in EC

DNA methylation is an epigenetic modification, and its overall reduction is closely related to the occurrence, development, and cellular carcinogenesis of tumors (35). We obtained the methylation level of the HMGA2 promoter in endometrial cancer and normal tissues from UALCAN. Compared to normal tissues, the methylation level of the HMGA2 promoter in endometrial cancer was significantly reduced, especially in stage III tumors, where the decrease was most pronounced, leading to reduced chromosomal stability and increased tumorigenesis (**Supplementary Figures 1A, B**).

To explore the interacting proteins and potential biological functions of HMGA2, we used the GeneMANIA to generate a protein interaction network diagram for HMGA2. As shown in **Supplementary Figure 1C**, HMGA2 was mainly co-expressed with HMGA1, E4F1, and UBN1, and these proteins were primarily involved in chromatin assembly and disassembly, DNA packaging, and transcription regulation. Then, GO and KEGG pathway analyses were conducted, consistently revealing the association of HMGA2 with cell proliferation and tumor related pathways (**Supplementary Figures 1D, E**).

### 3.3 The immune infiltration of HMGA2 in EC

**Figure 2:**
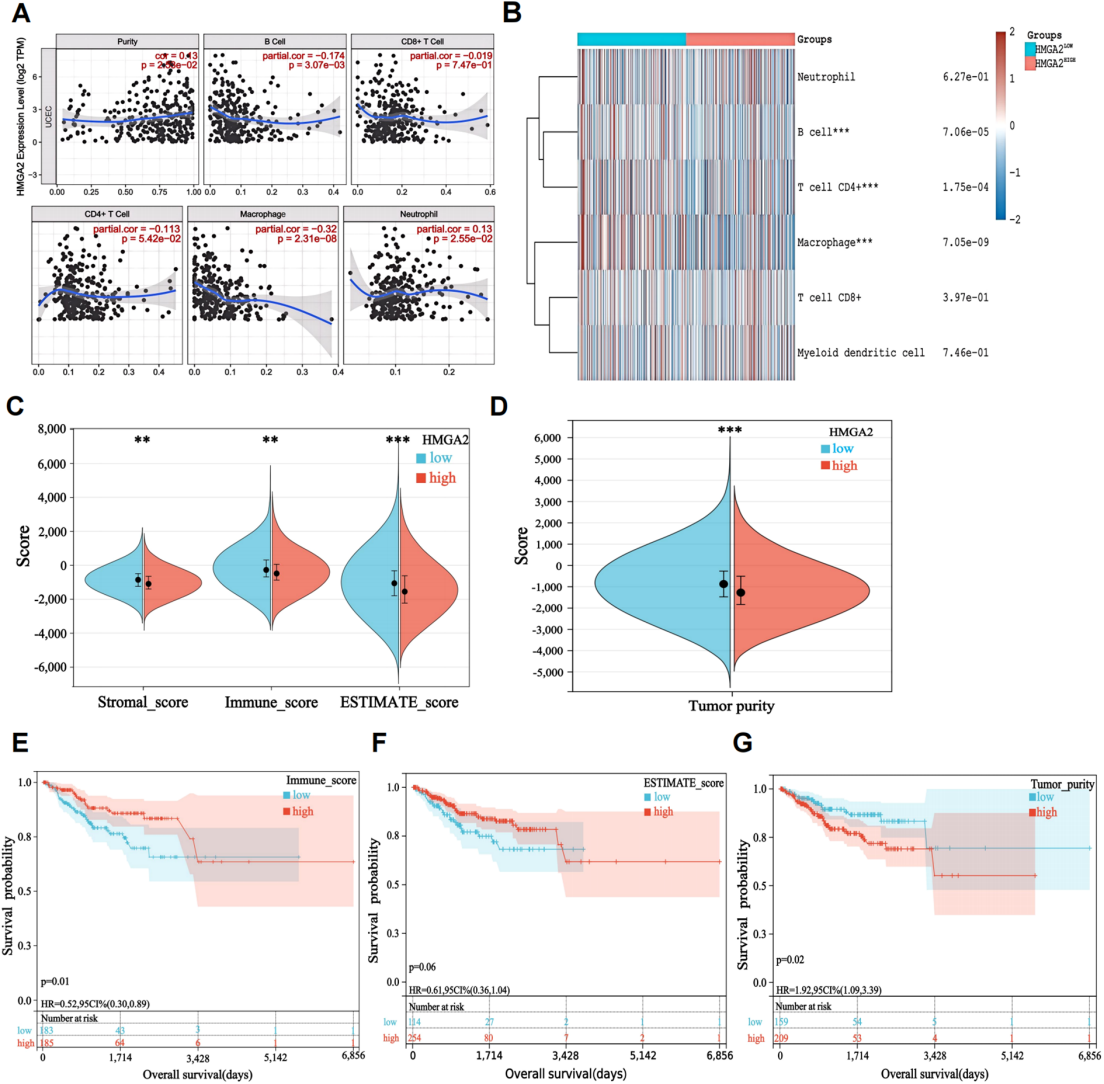
Characteristics of HMGA2 in immune cells infiltration. **(A)** Relationship between HMGA2 expression and immune infiltration level generated from TIMER. **(B)** Clustering of MCP‐counter scores for the correlation of HMGA2 with immune and non-immune stromal cell populations in UCEC according to TCGA databases. **(C, D)** Differential expression level of HMGA2 with immune scores, stromal scores, estimate scores and tumor purity in UCEC. **(E-G)** OS curves of different levels of immune scores, estimate scores and tumor purity. Notes: (**p < 0.01, ***p < 0.001, ns, not significant).

Immune infiltration dynamics demonstrated significant associations with tumor progression and clinical outcomes. Utilizing the TIMER database, we investigated HMGA2’s role in modulating the endometrial cancer immune microenvironment. We observed a negative correlation between high expression of HMGA2 and infiltration of most immune cells (B cells, T cells, macrophages, etc.), with the strongest negative correlation observed with macrophage infiltration (r=-0.32, P=2.31e-08; **Figures 2A, B**). ESTIMATE algorithm analysis further revealed significantly elevated stromal scores (P<0.01), immune scores (P<0.01), and ESTIMATE scores (P<0.001) alongside reduced tumor purity (P<0.001) in HMGA2-low tumors (all vs HMGA2-high; **Figures 2C, D**). Critically, superior progression-free survival was associated with heightened immune score (HR=0.52, P=0.01), ESTIMATE score (HR=0.61, P=0.06), and Tumor purity (HR=1.92, P=0.02), confirming that HMGA2-mediated immunosuppression may contribute to poor prognosis through inhibition of anti-tumor immunity (**Figures 2E–G**).

Building on evidence of HMGA2-mediated immunosuppression, we further explored the correlation between lymphocyte infiltration, immune checkpoint inhibitors, and immune checkpoint stimulators in EC with HMGA2. The results showed that HMGA2 was mainly negatively correlated with immune cell infiltration, including eosinophils, monocytes, macrophages, and activated B cells. Similarly, it was also significantly negatively correlated with immune checkpoint inhibitors and activators. Among them, we found that the expression of HMGA2 was significantly negatively correlated with the expression of M1 macrophage marker CD86 (r=-0.186, P=1.3e-05; **Supplementary Figure 2**). In summary, these findings suggested that patients with high HMGA2 expression levels may have less immune cell infiltration (especially M1 macrophages infiltration) at the tumor site and may be less responsive to immune checkpoint inhibitor therapy.

**Figure 3:**
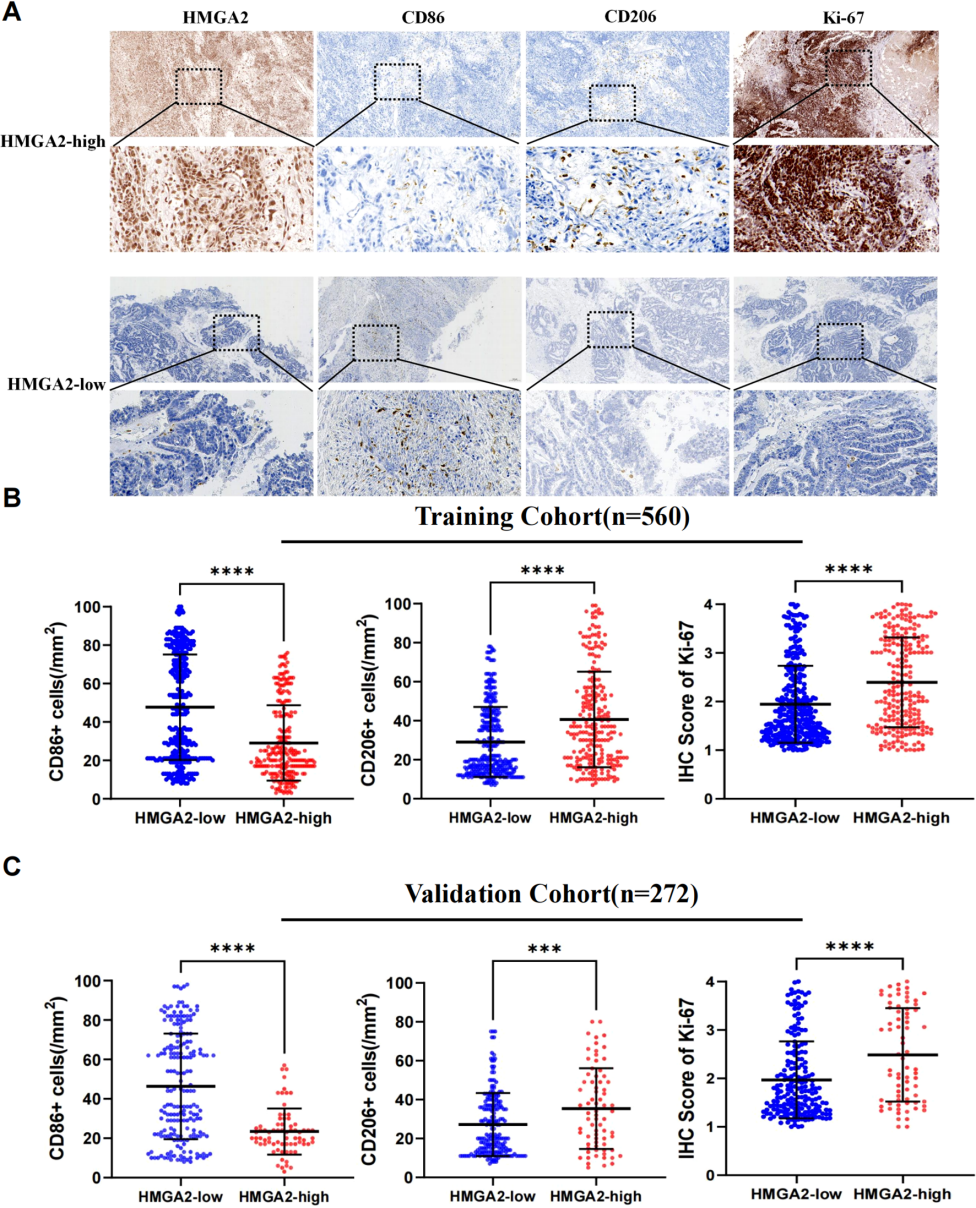
Immunohistochemical analysis in two cohorts. **(A)** Immunohistochemistry of HMGA2, CD86, CD206, Ki-67 expression in two cohorts. **(B)** Correlation between HMGA2 expression and CD86^+^ cells, CD206^+^ cells, IHC Score of Ki-67 in the training cohort. **(C)** Correlation between HMGA2 expression and CD86^+^ cells, CD206^+^ cells, IHC Score of Ki-67 in the validation cohort. Notes: (***p < 0.001, ****p < 0.0001, ns, not significant).

Mechanistic dissection through GSEA confirmed HMGA2’s central role in macrophage ontogeny, demonstrating coordinated enrichment in macrophage activation (NES = 2.01, P = 0.0), proliferation (NES = 1.79, P = 0.0), and differentiation (NES = 2.02, P = 0.0) (**Supplementary Figure 3**). Spatial validation in dual-center cohorts (training cohort n=560 and validation cohort n=262, the comparison of baseline data between the two cohorts was shown in **Supplementary Table 4**) via multiplex immunohistochemical analyses quantified HMGA2-driven protumorigenic remodeling: high-HMGA2 tumors exhibited elevated Ki-67 proliferative index (P<0.001), concomitant depletion of M1-like immunostimulatory macrophages (CD86^+^ cells, P<0.001) and expansion of M2-like immunosuppressive subsets (CD206^+^ cell, P<0.001) (**Figure 3**). This phenotypic switch of M2/M1 establishes HMGA2 as a pathological driver of macrophage repolarization toward immune-evasive niches.

### 3.4 HMGA2 modulated the proliferation, migration, and invasion of EC cells

**Figure 4:**
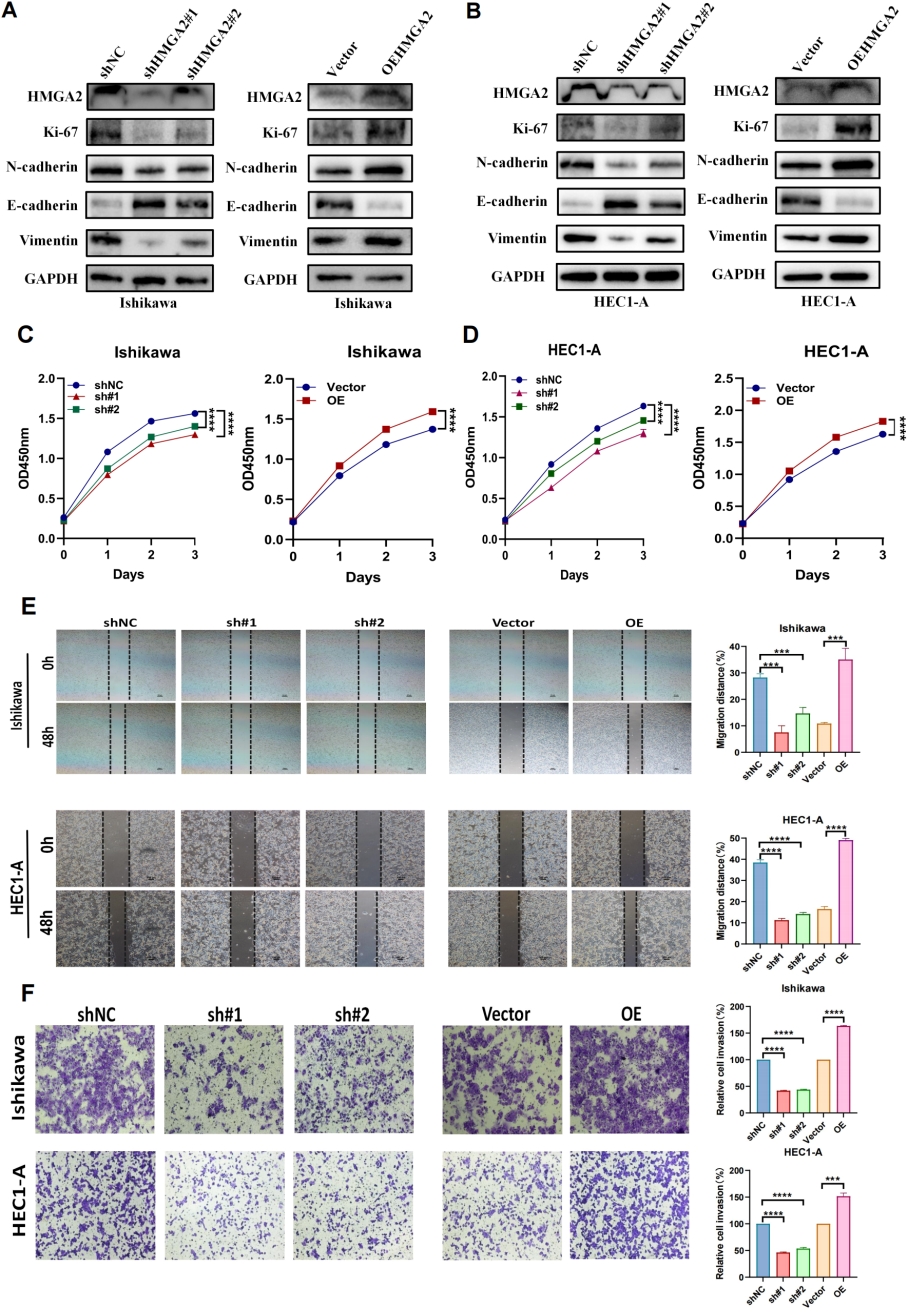
Knockdown of HMGA2 inhibits cell migration and proliferation. **(A, B)** WB analysis of the expression of HMGA2, EMT markers in Ishikawa or HEC1-A cells transfected with si-HMGA2 or OEHMGA2. **(C, D)** CCK-8 assay was performed to evaluate the proliferation of Ishikawa and HEC1-A cells after silencing HMGA2. **(E)** Scratch assays of Ishikawa and HEC1-A cells after transfection with si-HMGA2. **(F)** Transwell migration assays were performed after transfection with si-HMGA2 in Ishikawa and HEC1-A cells. Notes: (***p < 0.001, ****p < 0.0001, ns, not significant).

HMGA2 was significantly overexpressed in EC tissues. To define its functional impact, lentiviral-mediated transduction (pLKO.1-shHMGA2/pLVX-HMGA2) established Ishikawa and HEC1-A cell models with stable knockdown or overexpression (**Supplementary Figure 4**). Subsequent cellular functional assays demonstrated that HMGA2 knockdown significantly suppressed proliferation, migration and invasion. Synchronously, we found that after HMGA2 knockdown, the proliferation marker Ki-67 and migration related markers N-cadherin and Vimentin decreased, while the expression level of E-cadherin increased (**Figure 4**). Conversely, HMGA2 overexpression amplified oncogenic phenotypes proportionally.

**Figure 5:**
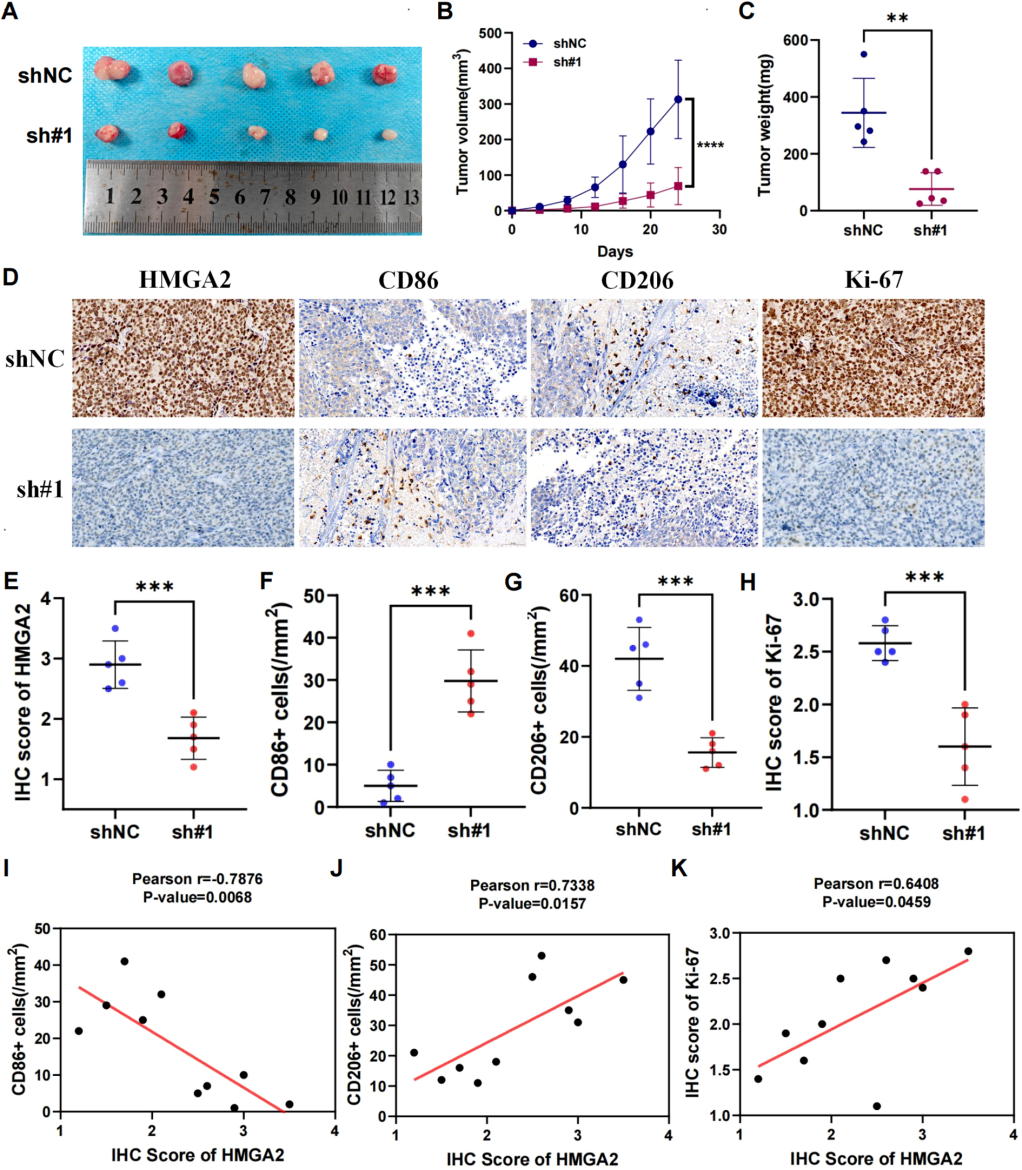
Knockdown of HMGA2 inhibits tumor growth and macrophage polarization in endometrial cancer *in vivo*. Ishikawa cells (1 × 10^7^) were injected subcutaneously into mice of the control group and shHMGA2#1 group. Each group contained 5 mice. **(A)** Images of the xenograft tumors from all mice at the endpoint. **(B)** The tumor volumes of mice. **(C)** The tumor weight of mice. **(D)** The levels of HMGA2, CD86, CD206, Ki-67 in tumor tissues were measured using Immunohistochemistry. **(E-H)** The levels of HMGA2, CD86, CD206, Ki-67 in the control group and shHMGA2#1 group. **(I-K)** The correlation between HMGA2 and CD86, CD206, Ki-67. Notes: (**p < 0.01, ***p < 0.001, ****p < 0.0001, ns, not significant).

The tumor-promoting role was corroborated *in vivo* using shRNA-transduced Ishikawa xenografts (BALB/c nude, n=5/group). shHMGA2#1 tumors exhibited 4.5-fold slower growth kinetics (**Figures 5A, B**: day24 volume 69.3 ± 52.1mm³ vs 312.9 ± 110.3 mm³, p<0.001) and 77.9% reduction in terminal tumor weight (**Figure 5C**: 76.2 ± 57.3mg vs 344.2 ± 121.4mg, p<0.01). Immunohistochemical profiling of xenografts revealed coordinated molecular alterations: HMGA2 silencing attenuated the expression of Ki-67 (IHC score 2.6 ± 0.2% vs 1.6 ± 0.4%, p<0.001), while modulating tumor-associated macrophages—CD86^+^ M1 density decreased by 83.2% (5.0 ± 3.7 vs 29.8 ± 7.3 cells/mm², p<0.001), whereas CD206^+^ M2 infiltration increased 2.7-fold (42.0 ± 8.9 vs 15.6 ± 4.2 cells/mm², p<0.001) (**Figures 5D–H**). Spearman analysis confirmed HMGA2 positively correlated with Ki-67 (r=0.6408, p=0.0459) and CD206 (r=0.7338, p=0.0157), but inversely with CD86 (r=-0.7876, p=0.0068), indicating its dual role in driving carcinogenesis and sculpting immunosuppressive niches (**Figures 5I–K**).

### 3.5 Chemotherapy and immunotherapy response of HMGA2 in EC

**Figure 6:**
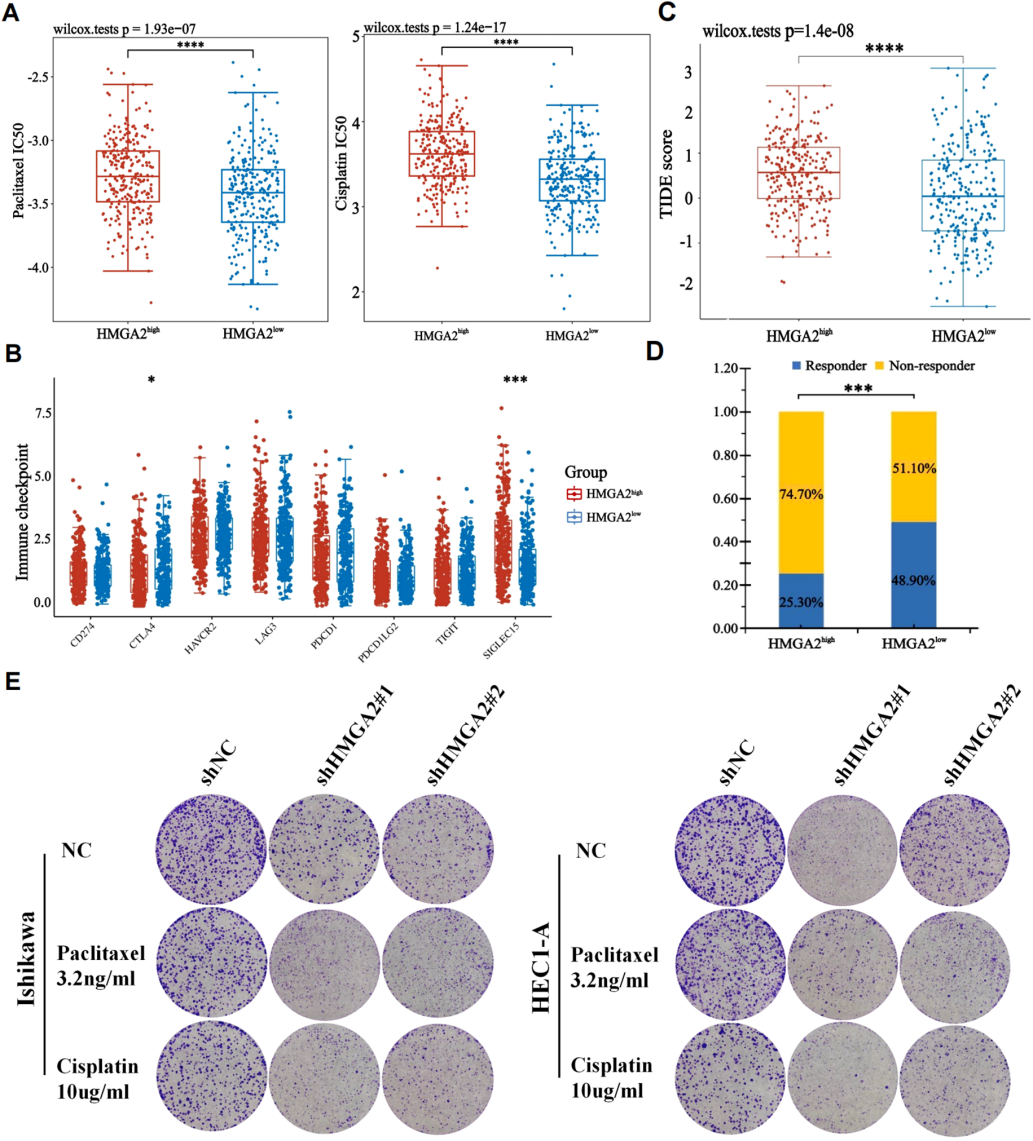
The correlation between HMGA2 expression and chemotherapy and immunotherapy. **(A)** The IC50 values of paclitaxel and cisplatin toxicity in differentially HMGA2 expressed groups. **(B)** Expression of immune checkpoint molecules related to HMGA2 differential expression in UCEC patients. **(C)** Prediction of immunotherapy response using the TIDE computational framework. **(D)** Comparison of populations in responders and non-responders to immunotherapy based on TIDE scores. **(E)** Colony formation assays upon HMGA2 knockdown in Ishikawa or HEC1-A cells after 2-week paclitaxel or cisplatin treatment. Notes: (*p < 0.05, ***p < 0.001, ****p < 0.0001, ns, not significant).

Given that most endometrial carcinomas are adenocarcinomas where immunotherapy-chemotherapy combinations constitute first-line therapy for advanced/recurrent disease (1), we pharmacologically profiled HMGA2 using GDSC database. High HMGA2 expression correlated with primary resistance to conventional chemotherapeutics (**Figure 6A**) and impaired responsiveness to immune checkpoint inhibitors (**Figure 6B**). Conversely, the HMGA2-low cohort exhibited enhanced drug sensitivity and superior immunotherapy outcomes (**Figures 6C, D**). Mechanistically, this chemoresistance and immune evasion phenocopied the HMGA2-induced immunosuppressive microenvironment characterized by CD206^+^ TAM expansion and CD86^+^ depletion, implicating distinct therapeutic implications: HMGA2-high tumors are candidates for targeted pathway inhibition, whereas HMGA2-low tumors may derive maximal benefit from immunotherapeutic approaches.

**Figure 7:**
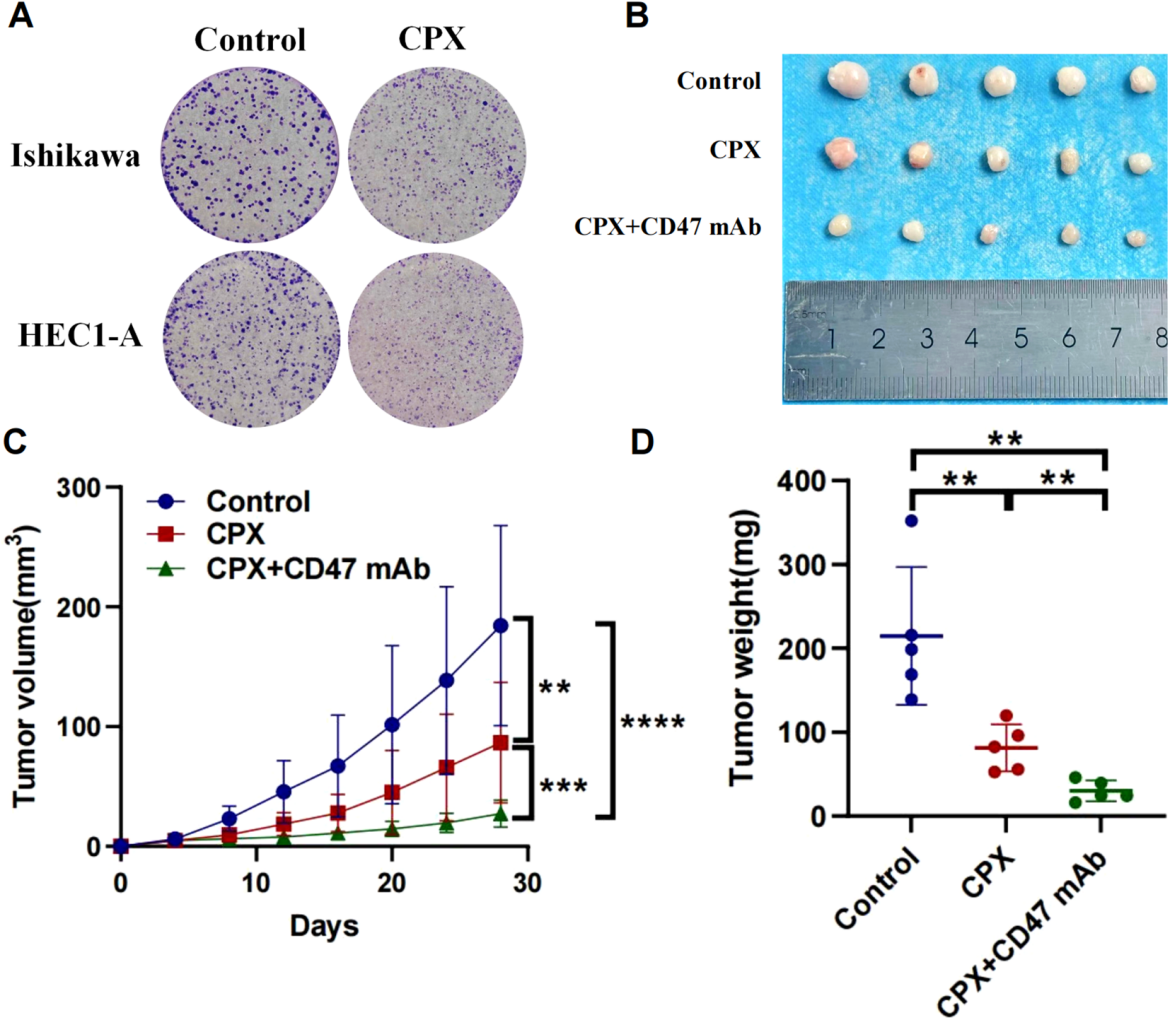
Anti-tumor effects of targeting HMGA2 (CPX) and CD47 in endometrial carcinoma. **(A)** Colony formation assays in Ishikawa or HEC1-A cells after 2-week CPX(10μM). **(B-D)** In a BALB/c nude mouse model with subcutaneous implantation of Ishikawa cells (1 × 10^7^), tumor photographs, tumor volume, and tumor weight were assessed following: Oral administration of CPX (100 mg/kg) three times daily; Oral administration of CPX (100 mg/kg) three times daily combined with intraperitoneal injection of anti-human CD47 monoclonal antibody (1 mg/kg) twice weekly. Notes: (**p < 0.01, ***p < 0.001, ****p < 0.0001, ns, not significant).

Complementary to pharmacogenomic predictions, HMGA2 depletion potentiated the cytotoxic effects of conventional chemotherapeutics in endometrial cancer models. Colony formation assays revealed that HMGA2 knockdown significantly augmented paclitaxel/cisplatin efficacy, the number of clones has significantly decreased compared to the control group (**Figure 6E**), while HMGA2 overexpression completely reversed the aforementioned effects (**Supplementary Figure 5**), confirming HMGA2-driven chemoresistance. *In vitro* cloning experiments, the application of HMGA2 inhibitors significantly inhibited the colony formation of EC cells (**Figure 7A**), while in xenograft models (Ishikawa/BALB/c nude), HMGA2 pharmacologic inhibition (po. 100 mg/kg three times daily) significantly suppressed tumor growth versus vehicle (53.1% volume reduction; p=0.01) (**Figure 7B**). Strikingly, combinatorial blockade of HMGA2 and CD47 checkpoint (anti-CD47 mAb 1 mg/kg biweekly, the main function is to enhance the phagocytic activity of macrophages towards tumor cells) synergistically amplified antitumor efficacy, achieving near-complete regression (85.1% suppression; p<0.001 vs monotherapies), establishing dual-targeting strategy as a promising therapeutic paradigm (**Figures 7B–D**).

### 3.6 The model for predicting EC recurrence based on HMGA2 and clinical pathological parameters

**Figure 8:**
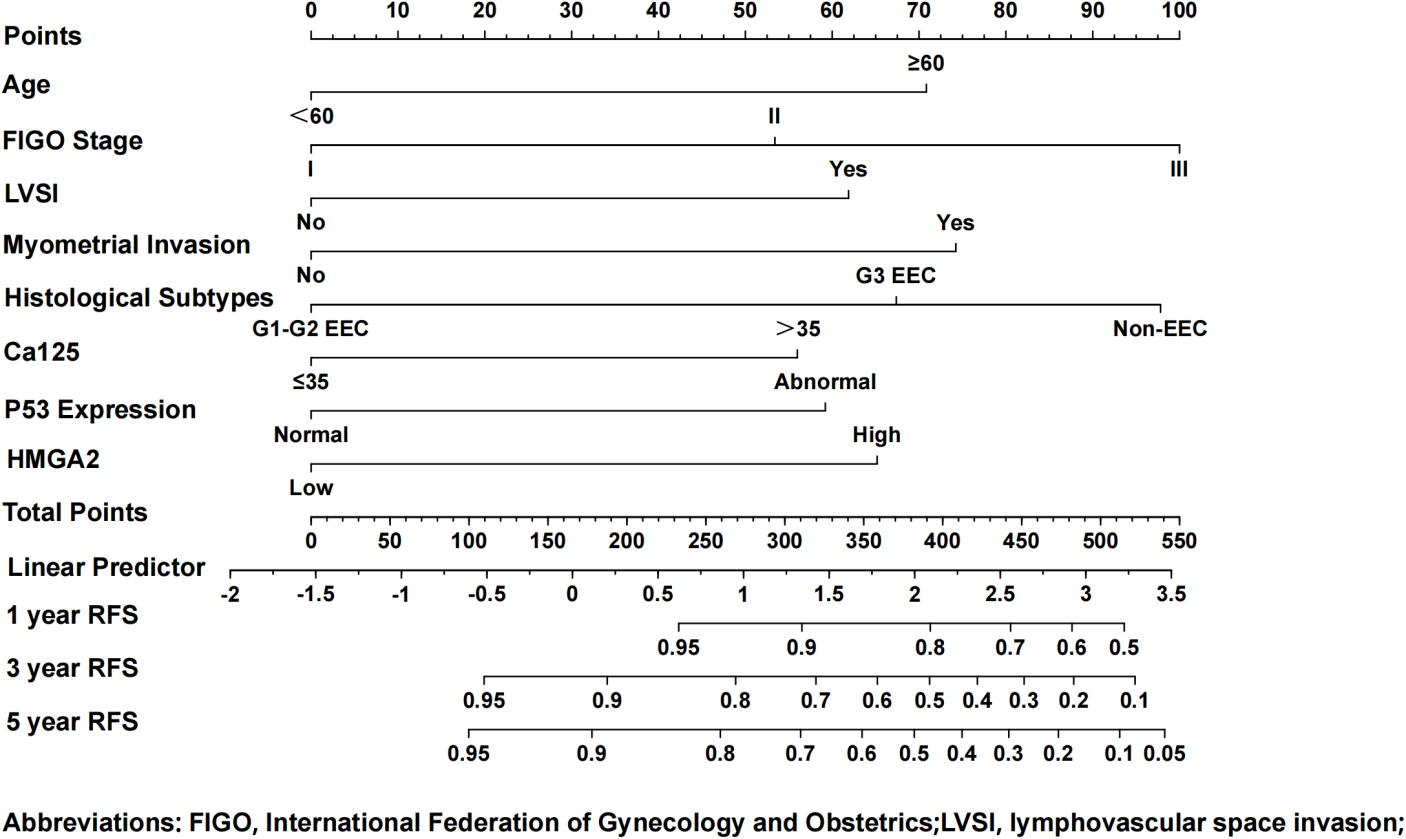
Nomogram model for predicting the 1-, 3-, and 5-year RFS rates of endometrial cancer patients. To predict the 1-, 3-, and 5-year RFS rates of endometrial cancer patients, draw the vertical line segment to the “Points” axis to get the corresponding score of each predictor, and calculate the total score of all predictors. Draw the vertical line segment from the “Total Points” axis to the “1-year RFS”, “3-year RFS”, and “5-year RFS” axis to get the corresponding 1-year, 3-year and 5-year RFS rates of endometrial cancer patients.

We further explored the clinical application value of HMGA2 in EC. Firstly, in the training cohort, HMGA2 emerged as an independent recurrence predictor on multivariate Cox regression after adjusting clinicopathological confounders. Alongside established risk factors (age, FIGO stage, LVSI, CA125, myometrial invasion, histological subtypes, P53; all P<0.05), HMGA2 overexpression significantly correlated with reduced RFS (HR=1.603, 95%CI 1.012–2.540; P=0.044) (**Supplementary Table 5**). The integrated prognostic model incorporating HMGA2 demonstrated superior discriminative power (AUC: 0.855 vs 0.593 for HMGA2 alone, 0.812 for clinical factors; **Supplementary Figure 6**), with HMGA2 contributing a large weight on nomogram among 8 variables (**Figure 8**).

**Figure 9:**
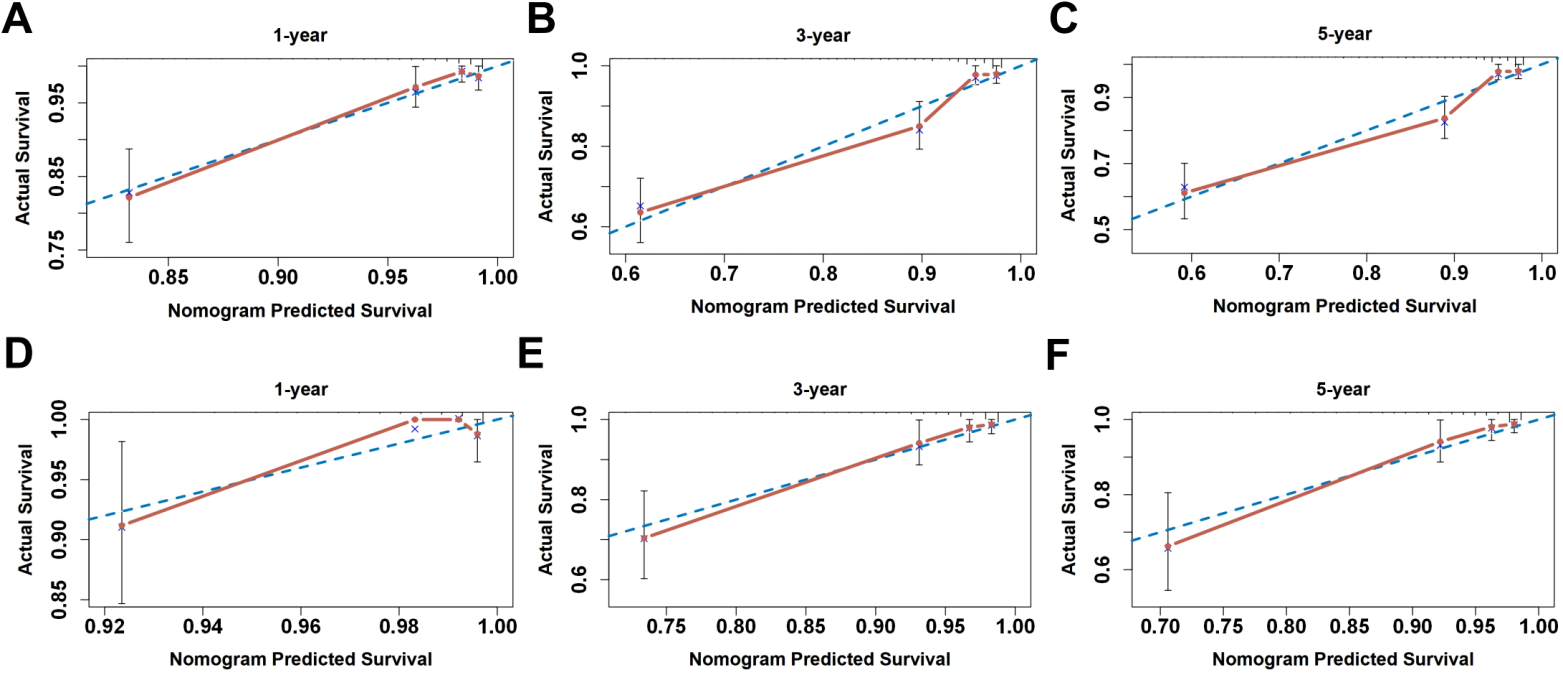
The calibration curve for internal and external validation of the nomogram model. **(A–C)** The internal calibration curve and **(D–F)** the external calibration curve of the nomogram for predicting the 1-, 3-, and 5-year RFS rates of endometrial cancer patients, respectively.

The nomogram demonstrated high calibration accuracy, with internal and external calibration curves at 1/3/5-year intervals showing strong agreement between predicted and observed recurrence-free probabilities (**Figure 9**). ROC curve and the maximum Youden index indicated that the optimal threshold for the nomogram model predicting 3-year recurrence of endometrial cancer was 0.868 (**Supplementary Figure 7**). Based on this threshold, we stratified patients into clinically distinct risk cohorts: High-risk recurrence group (nomogram score ≤0.868) and Low-risk recurrence group (>0.868). Kaplan-Meier analysis confirmed significant survival disparity, with consistent outcomes across both cohorts (detailed rates in **Supplementary Figure 8**).

## 4 Discussion

HMGA2, as a non-histone transcription factor, can influence various biological processes. The overexpression of HMGA2 is a characteristic of malignant tumors (36). For example, HMGA2 mediated the occurrence of triple-negative breast cancer by activating the NF-kB/IL-6/IL-8/STAT3 axis (37); HMGA2 activated the mTOR signaling pathway to inhibit ferroptosis, thereby enhancing the death resistance of pancreatic cancer and reducing chemotherapy sensitivity (38). Some studies have also shown that high expression of HMGA2 weakened the inhibitory effect of miR-302a-5p/367-3p on the malignant behavior of endometrial cancer cells, thereby promoting the progression of EC and being associated with poor prognosis in EC patients (9).

In this study, we utilized bioinformatics techniques and real clinical data to explore the differential expression and prognostic levels of HMGA2 in endometrial cancer. The results indicated that HMGA2 expression was significantly upregulated in endometrial cancer, was significantly associated with poor clinical and pathological features of EC, and that patients with high HMGA2 expression had significantly decreased overall survival and disease-free survival rates. These suggested that HMGA2 had good diagnostic and prognostic predictive value in endometrial cancer. KEGG enrichment analysis showed that HMGA2 was associated with tumor pathways such as cell cycle regulation, cell proliferation, Hippo signaling pathway, and TGF−beta signaling pathway. Functional experiments also indicated that knocking down HMGA2 significantly inhibited the proliferation, migration, and invasion of EC tumor cells. These results collectively confirm its dual role as prognostic biomarker and therapeutic target.

Existing studies have shown that HMGA2 interacted with certain immune cells. Xu et al. confirmed that HMGA2 reduced the sensitivity of colorectal cancer (CRC) cells to CD8+-T cell-mediated cytotoxicity through the miRNA-200c-3p/LSAMP/Wnt axis, promoting tumor development (39). The overexpression of HMGA2 in CRC cells promoted macrophage recruitment and M2 polarization by upregulating STAT3-mediated CCL2 secretion, thereby promoting tumor immune suppression in CRC (40). However, there are few studies reporting on the relationship between HMGA2 and immune infiltration in EC. In this study, we explored the relationship between HMGA2 and immune infiltration. Significant inverse correlation between HMGA2 expression and immune infiltration, particularly macrophage depletion. The immunohistochemical analysis results of our patients showed that high HMGA2 associated with reduced M1 marker CD86 yet elevated M2 marker CD206. At the same time, the efficacy of immune checkpoint inhibitors was significantly negatively correlated with HMGA2 expression. These findings suggested that HMGA2 may reprogram tumor-associated macrophages towards an immunosuppressive M2 phenotype in EC. Consequently, patients with high HMGA2 exhibit primary immunotherapy resistance, whereas low expressors are potential responders. Notably, dual targeting strategies demonstrate promise: HMGA2 depletion sensitizes tumors to chemotherapy, while combining HMGA2/CD47 blockade synergistically enhances anti-tumor efficacy.

Finally, to determine the prognostic significance of HMGA2 in EC, univariate and multivariate Cox regression analyses identified HMGA2 as an independent predictor of recurrence. Integrating HMGA2 with key clinicopathological parameters yielded a novel prognostic model demonstrating superior predictive accuracy compared to conventional models. Stratifying patients into high- and low-recurrence risk groups at the model’s optimal threshold revealed significantly worse survival outcomes in the high-risk group. This necessitates intensified postoperative follow-up and multimodal therapeutic approaches for high-risk patients, who were predominantly characterized by high HMGA2 expressions. Notably, elevated HMGA2 levels correlated with suppressed immune infiltration, suggesting HMGA2-high patients may benefit from targeted HMGA2 inhibition, whereas HMGA2-low patients, potentially more immunotherapy-sensitive, might derive greater advantage from immunotherapeutic strategies.

This study suggests that HMGA2 promotes endometrial carcinogenesis and progression, potentially by modulating immune infiltration levels, highlighting its value as a biomarker. However, a notable limitation is the lack of in-depth investigation into the precise mechanisms by which HMGA2 influences macrophage differentiation, which should be elucidated in future studies.

## 5 Conclusion

In summary, this study demonstrates that HMGA2 expression is associated with poor prognosis in endometrial cancer (EC), promoting disease progression potentially by inhibiting M1-polarized macrophage differentiation. Furthermore, patients exhibiting low HMGA2 levels showed enhanced sensitivity to immunotherapy. These findings underscore HMGA2’s potential utility as both a prognostic biomarker and a predictor of immunotherapy response. The development of models incorporating HMGA2 expression offers a valuable tool for risk stratification and has significant potential for informing clinical decision-making regarding treatment selection.

## Data Availability

The original contributions presented in the study are included in the article/**Supplementary Material**. Further inquiries can be directed to the corresponding authors.

## References

[1] Amant F Moerman P Neven P Timmerman D Van Limbergen E Vergote I. Endometrial cancer. Lancet. (2005) 366:491–505. doi: 10.1016/S0140-6736(05)67063-8, PMID: 16084259

[2] Thanapprapasr D Cheewakriangkrai C Likittanasombut P Thanapprapasr K Mutch DG. Targeted endometrial cancer therapy as a future prospect. Womens Health (Lond). (2013) 9:189–99. doi: 10.2217/WHE.13.4, PMID: 23477324

[3] van den Heerik A Horeweg N de Boer SM Bosse T Creutzberg CL. Adjuvant therapy for endometrial cancer in the era of molecular classification: radiotherapy, chemoradiation and novel targets for therapy. Int J Gynecol Cancer. (2021) 31:594–604. doi: 10.1136/ijgc-2020-001822, PMID: 33082238 PMC8020082

[4] Mansoori B Mohammadi A Ditzel HJ Duijf PHG Khaze V Gjerstorff MF. HMGA2 as a critical regulator in cancer development. Genes (Basel). (2021) 12:269. doi: 10.3390/genes12020269, PMID: 33668453 PMC7917704

[5] Thanos D Maniatis T. The high mobility group protein HMG I(Y) is required for NF-kappa B-dependent virus induction of the human IFN-beta gene. Cell. (1992) 71:777–89. doi: 10.1016/0092-8674(92)90554-P, PMID: 1330326

[6] Gao X Dai M Li Q Wang Z Lu Y Song Z. HMGA2 regulates lung cancer proliferation and metastasis. Thorac Cancer. (2017) 8:501–10. doi: 10.1111/1759-7714.12476, PMID: 28752530 PMC5582513

[7] Malek A Bakhidze E Noske A Sers C Aigner A Schäfer R. HMGA2 gene is a promising target for ovarian cancer silencing therapy. Int J Cancer. (2008) 123:348–56. doi: 10.1002/ijc.23491, PMID: 18452175

[8] Mansoori B Duijf PHG Mohammadi A Najafi S Roshani E Shanehbandi D. Overexpression of HMGA2 in breast cancer promotes cell proliferation, migration, invasion and stemness. Expert Opin Ther Targets. (2020) 24:255–65. doi: 10.1080/14728222.2020.1736559, PMID: 32172636

[9] Ma J Li D Kong FF Yang D Yang H Ma XX. miR-302a-5p/367-3p-HMGA2 axis regulates Malignant processes during endometrial cancer development. J Exp Clin Cancer Res. (2018) 37:19. doi: 10.1186/s13046-018-0686-6, PMID: 29391048 PMC5796297

[10] Lin L Lin G Lin H Chen L Chen X Lin Q. Integrated profiling of endoplasmic reticulum stress-related DERL3 in the prognostic and immune features of lung adenocarcinoma. Front Immunol. (2022) 13:906420. doi: 10.3389/fimmu.2022.906420, PMID: 36275646 PMC9585215

[11] Guo L Li F Liu H Kong D Chen C Sun S. SIX1 amplification modulates stemness and tumorigenesis in breast cancer. J Transl Med. (2023) 21:866. doi: 10.1186/s12967-023-04679-2, PMID: 38031089 PMC10685563

[12] Warde-Farley D Donaldson SL Comes O Zuberi K Badrawi R Chao P. The GeneMANIA prediction server: biological network integration for gene prioritization and predicting gene function. Nucleic Acids Res. (2010) 38:W214–20. doi: 10.1093/nar/gkq537, PMID: 20576703 PMC2896186

[13] Cerami E Gao J Dogrusoz U Gross BE Sumer SO Aksoy BA. The cBio cancer genomics portal: an open platform for exploring multidimensional cancer genomics data. Cancer Discov. (2012) 2:401–4. doi: 10.1158/2159-8290.CD-12-0095, PMID: 22588877 PMC3956037

[14] Zhou Y Zhou B Pache L Chang M Khodabakhshi AH Tanaseichuk O. Metascape provides a biologist-oriented resource for the analysis of systems-level datasets. Nat Commun. (2019) 10:1523. doi: 10.1038/s41467-019-09234-6, PMID: 30944313 PMC6447622

[15] Madikyzy M Tilegen M Nazarbek G Mu C Kutzhanova A Li X. Honghua extract mediated potent inhibition of COVID-19 host cell pathways. Sci Rep. (2022) 12:14296. doi: 10.1038/s41598-022-15338-9, PMID: 35995784 PMC9395372

[16] Wu W Wang X Le W Lu C Li H Zhu Y. Immune microenvironment infiltration landscape and immune-related subtypes in prostate cancer. Front Immunol. (2022) 13:1001297. doi: 10.3389/fimmu.2022.1001297, PMID: 36700224 PMC9868452

[17] Yoshihara K Shahmoradgoli M Martínez E Vegesna R Kim H Torres-Garcia W. Inferring tumour purity and stromal and immune cell admixture from expression data. Nat Commun. (2013) 4:2612. doi: 10.1038/ncomms3612, PMID: 24113773 PMC3826632

[18] Ru B Wong CN Tong Y Zhong JY Zhong SSW Wu WC. TISIDB: an integrated repository portal for tumor-immune system interactions. Bioinformatics. (2019) 35:4200–2. doi: 10.1093/bioinformatics/btz210, PMID: 30903160

[19] Yang W Soares J Greninger P Edelman EJ Lightfoot H Forbes S. Genomics of Drug Sensitivity in Cancer (GDSC): a resource for therapeutic biomarker discovery in cancer cells. Nucleic Acids Res. (2013) 41:D955–61. doi: 10.1093/nar/gks1111, PMID: 23180760 PMC3531057

[20] Subramanian A Tamayo P Mootha VK Mukherjee S Ebert BL Gillette MA. Gene set enrichment analysis: a knowledge-based approach for interpreting genome-wide expression profiles. Proc Natl Acad Sci U S A. (2005) 102:15545–50. doi: 10.1073/pnas.0506580102, PMID: 16199517 PMC1239896

[21] Pecorelli S. Revised FIGO staging for carcinoma of the vulva, cervix, and endometrium. Int J Gynaecol Obstet. (2009) 105:103–4. doi: 10.1016/j.ijgo.2009.02.012, PMID: 19367689

[22] Jiang P Wang J Gong C Yi Q Zhu M Hu Z. A nomogram model for predicting recurrence of stage I-III endometrial cancer based on inflammation-immunity-nutrition score (IINS) and traditional classical predictors. J Inflammation Res. (2022) 15:3021–37. doi: 10.2147/JIR.S362166, PMID: 35645577 PMC9135581

[23] Jiang P Yuan R. Analysis of factors related to lymph node metastasis in early-stage type 1 endometrial cancer: verifying the clinical value of positive threshold of the immunohistochemical parameter ki67. Cancer Manag Res. (2021) 13:6319–28. doi: 10.2147/CMAR.S316211, PMID: 34413681 PMC8369284

[24] Fu YP Yi Y Cai XY Sun J Ni XC He HW. Overexpression of interleukin-35 associates with hepatocellular carcinoma aggressiveness and recurrence after curative resection. Br J Cancer. (2016) 114:767–76. doi: 10.1038/bjc.2016.47, PMID: 27002937 PMC4984866

[25] Jiang P Jia M Hu J Huang Z Deng Y Lai L. Prognostic value of ki67 in patients with stage 1–2 endometrial cancer: validation of the cut-off value of ki67 as a predictive factor. Onco Targets Ther. (2020) 13:10841–50. doi: 10.2147/OTT.S274420, PMID: 33149602 PMC7602913

[26] Zhou X Nie M Xin X Hua T Zhang J Shi R. RAB17 promotes endometrial cancer progression by inhibiting TFRC-dependent ferroptosis. Cell Death Dis. (2024) 15:655. doi: 10.1038/s41419-024-07013-w, PMID: 39242574 PMC11379720

[27] Liao S Yang Y Chen S Bi Y Huang Q Wei Z. IL-24 inhibits endometrial cancer cell proliferation by promoting apoptosis through the mitochondrial intrinsic signaling pathway. BioMed Pharmacother. (2020) 124:109831. doi: 10.1016/j.biopha.2020.109831, PMID: 31972354

[28] Zhang D Liang P Xia B Wu J Hu X. Comprehensive pan-cancer analysis of ZNF337 as a potential diagnostic, immunological, and prognostic biomarker. BMC Cancer. (2024) 24:987. doi: 10.1186/s12885-024-12703-x, PMID: 39123194 PMC11313096

[29] Wang L Li S Luo H Lu Q Yu S. PCSK9 promotes the progression and metastasis of colon cancer cells through regulation of EMT and PI3K/AKT signaling in tumor cells and phenotypic polarization of macrophages. J Exp Clin Cancer Res. (2022) 41:303. doi: 10.1186/s13046-022-02477-0, PMID: 36242053 PMC9563506

[30] Lin Y Chen X Lin L Xu B Zhu X Lin X. Sesamolin serves as an MYH14 inhibitor to sensitize endometrial cancer to chemotherapy and endocrine therapy via suppressing MYH9/GSK3β/β-catenin signaling. Cell Mol Biol Lett. (2024) 29:63. doi: 10.1186/s11658-024-00583-9, PMID: 38698330 PMC11067147

[31] Kong C Zhu Z Li Y Xue P Chen L. Downregulation of HOXA11 enhances endometrial cancer Malignancy and cisplatin resistance via activating PTEN/AKT signaling pathway. Clin Transl Oncol. (2021) 23:1334–41. doi: 10.1007/s12094-020-02520-6, PMID: 33515421

[32] Huang YM Cheng CH Pan SL Yang PM Lin DY Lee KH. Gene expression signature-based approach identifies antifungal drug ciclopirox as a novel inhibitor of HMGA2 in colorectal cancer. Biomolecules. (2019) 9:688. doi: 10.3390/biom9110688, PMID: 31684108 PMC6920845

[33] Zhang X Su T Wu Y Cai Y Wang L Liang C. N6-methyladenosine reader YTHDF1 promotes stemness and therapeutic resistance in hepatocellular carcinoma by enhancing NOTCH1 expression. Cancer Res. (2024) 84:827–40. doi: 10.1158/0008-5472.CAN-23-1916, PMID: 38241695

[34] Hu LY Zhuang WT Chen MJ Liao J Wu DF Zhang YX. EGFR oncogenic mutations in NSCLC impair macrophage phagocytosis and mediate innate immune evasion through up-regulation of CD47. J Thorac Oncol. (2024) 19:1186–200. doi: 10.1016/j.jtho.2024.03.019, PMID: 38553005

[35] Nishiyama A Nakanishi M. Navigating the DNA methylation landscape of cancer. Trends Genet. (2021) 37:1012–27. doi: 10.1016/j.tig.2021.05.002, PMID: 34120771

[36] Zhang S Mo Q Wang X. Oncological role of HMGA2 (Review). Int J Oncol. (2019) 55:775–88. doi: 10.3892/ijo.2019.4856, PMID: 31432151

[37] Mansoori B Terp MG Mohammadi A Pedersen CB Ditzel HJ Baradaran B. HMGA2 supports cancer hallmarks in triple-negative breast cancer. Cancers (Basel). (2021) 13:5197. doi: 10.3390/cancers13205197, PMID: 34680349 PMC8533747

[38] Luo Z Zheng Q Ye S Li Y Chen J Fan C. HMGA2 alleviates ferroptosis by promoting GPX4 expression in pancreatic cancer cells. Cell Death Dis. (2024) 15:220. doi: 10.1038/s41419-024-06592-y, PMID: 38493165 PMC10944463

[39] Xu X Gong C Wang Y Yin Z Wang X Hu Y. Bioinformatics analysis and experimental validation identified HMGA2/microRNA-200c-3p/LSAMP/Wnt axis as an immunological factor of patients with colorectal cancer. Am J Cancer Res. (2023) 13:3898–920., PMID: 37818072 PMC10560921

[40] Wang X Wang J Zhao J Wang H Chen J Wu J. HMGA2 facilitates colorectal cancer progression via STAT3-mediated tumor-associated macrophage recruitment. Theranostics. (2022) 12:963–75. doi: 10.7150/thno.65411, PMID: 34976223 PMC8692921

