# Supplementary material for: Prognostic significance and immune infiltration analysis of HMGA2 in endometrial cancer
Source: Front Immunol. 2025 Jul 9;16:1559278. doi: 10.3389/fimmu.2025.1559278 (PMC12283574; doi:10.3389/fimmu.2025.1559278)
Supplement: Supplementary file 1 [file DataSheet1.pdf]

Supplementary Figures and Tables

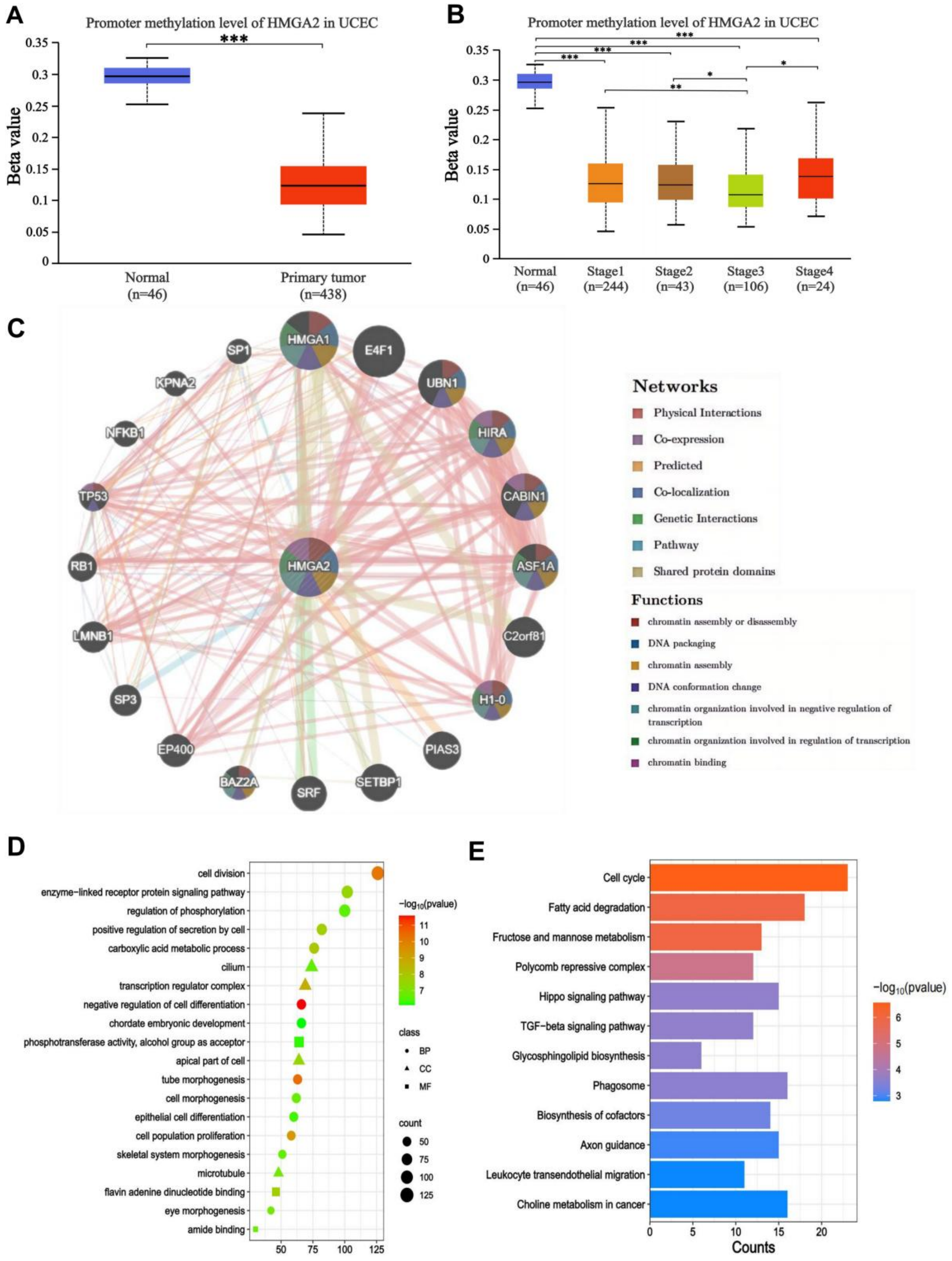

Supplementary Figure 1 Promoter methylation and functional annotation of HMGA2 in UCEC patients

(A) The differential methylation of the HMGA2 promoter in primary UCEC tumors.

(B) The correlation between HMGA2 promoter methylation and clinical stage of

UCEC patients. (C) HMGA2 co-expression network constructed by GeneMANIA. (D, E) GO and KEGG enrichment analysis of the co-expressed genes of HMGA2. (* $p < 0.05$, ** $p < 0.01$, *** $p < 0.001$).

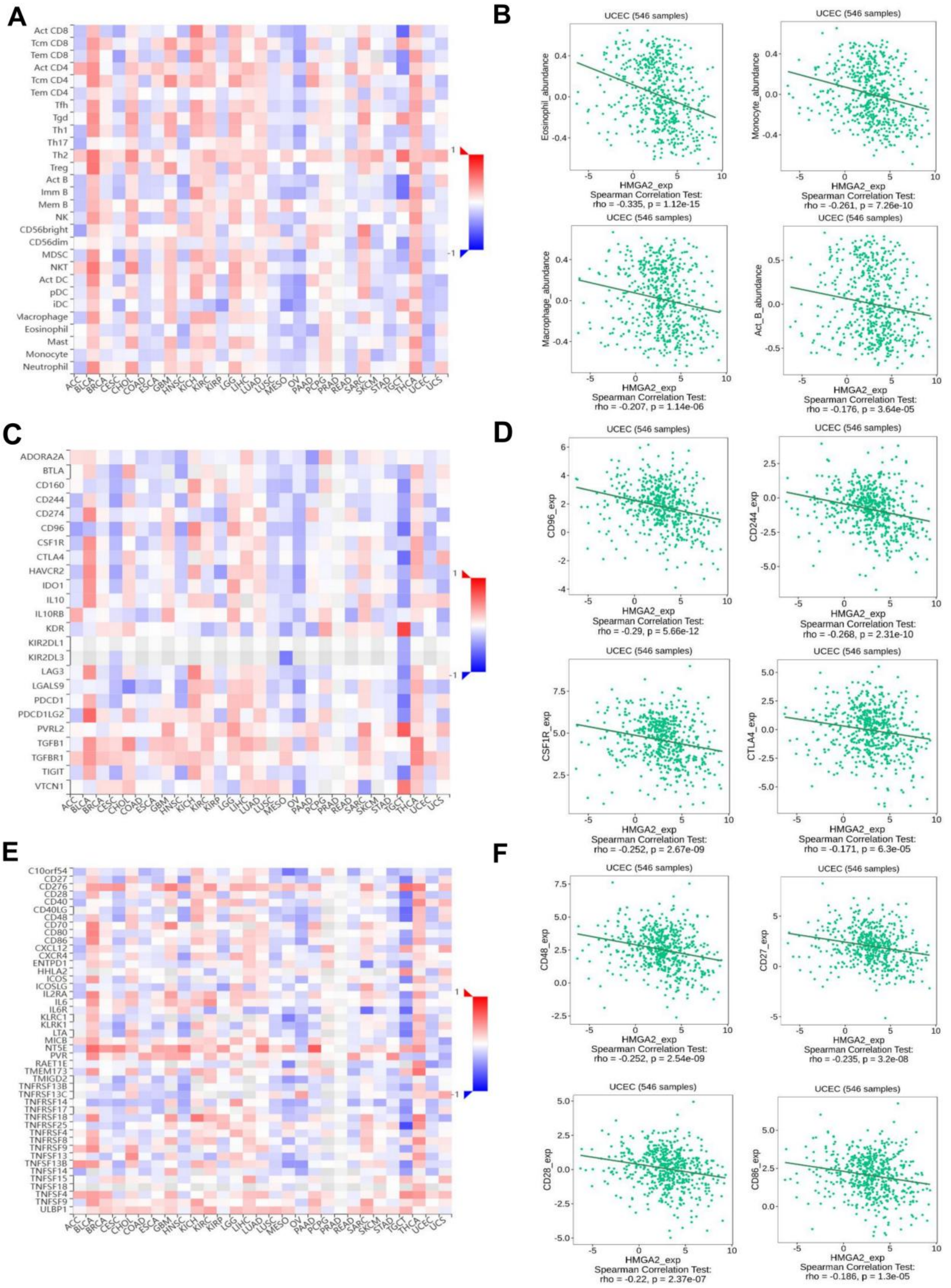

Supplementary Figure 2 The relationship between HMGA2 and the tumor microenvironment

(A) Relations with tumor-infiltrating lymphocytes in cancers, (B)and the main four results in UCEC. (C) Relations with immune inhibitors in cancers, (D) and the four main correlations in UCEC. (E) Relations with stimulators in cancers, (F) and the main four results in UCEC.

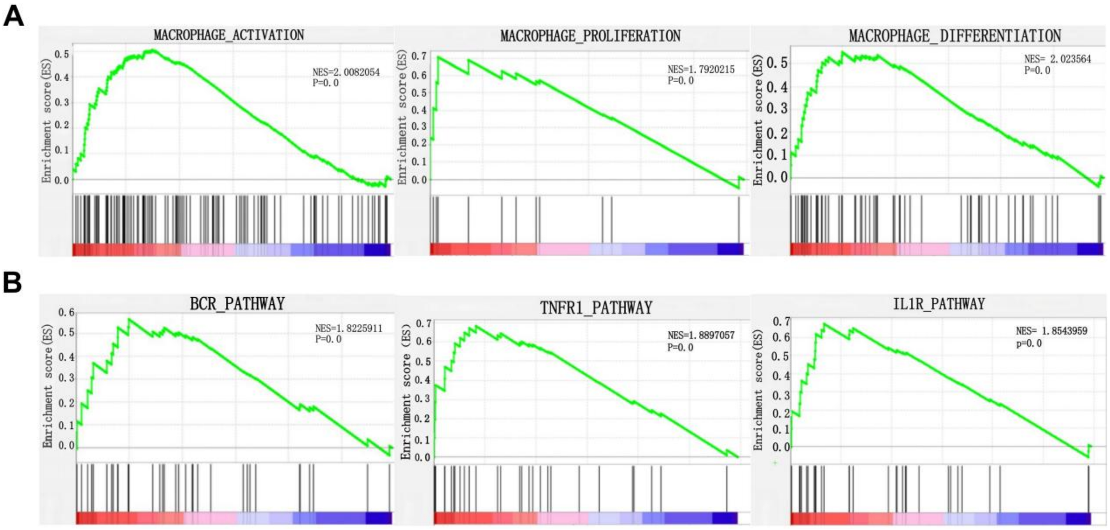

Supplementary Figure 3 Mechanisms underlying a HMGA2-mediated biological process in UCEC

(A, B) GSEA was performed using TCGA datasets. The macrophage activation, proliferation, and differentiation were identified with the strongest association with HMGA2 expression.

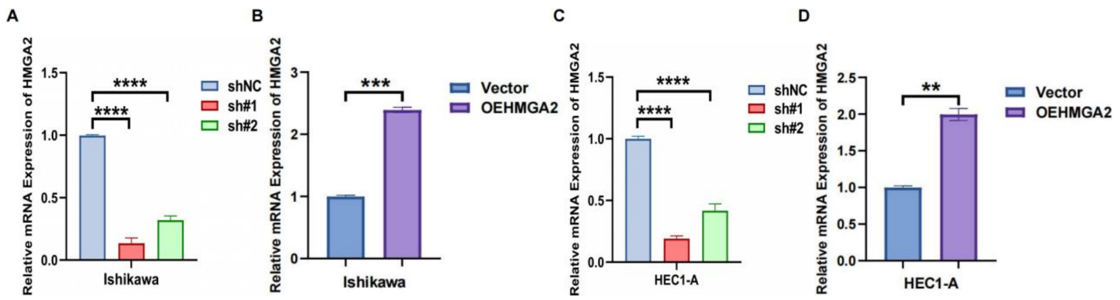

Supplementary Figure 4 Validation of HMGA2 transfection efficiency by qRT-PCR

(A) and (C) verification of knockdown efficiency of HMGA2 in Ishikawa and HEC1-A cells by qRT-PCR. (B) and (D) verification of overexpression efficiency of HMGA2 in Ishikawa and HEC1-A cells by qRT-PCR. Notes: *p < 0.05, **p < 0.01, ***p < 0.001, ****p < 0.0001.

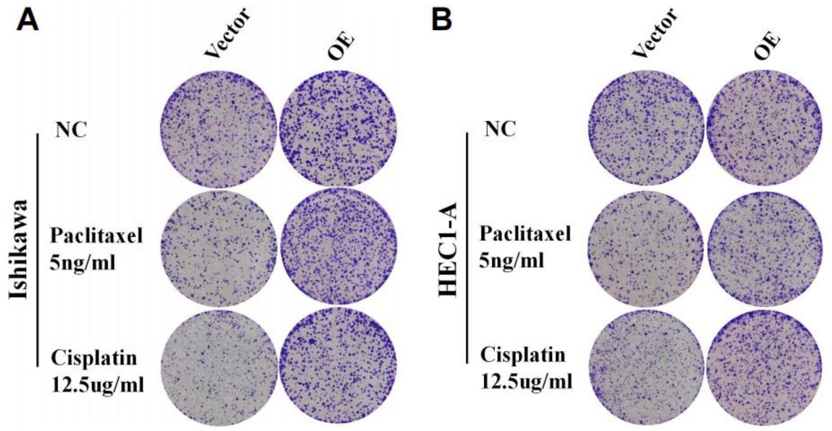

Supplementary Figure 5. The correlation between HMGA2 expression and chemotherapy

(A)Colony formation assays in HMGA2-overexpressing Ishikawa or HEC1-A cells after 2-week paclitaxel or cisplatin treatment.

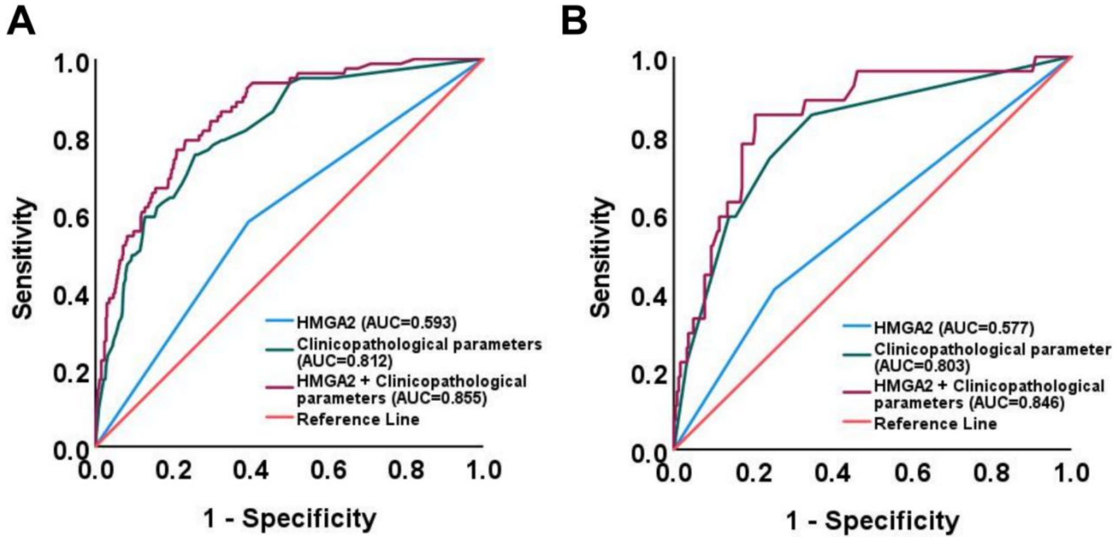

Supplementary Figure 6 Area under the curve (AUC) for HMGA2, clinicopathological parameters and their combination

(A)AUC in the training cohort. (B) AUC in the validation cohort.

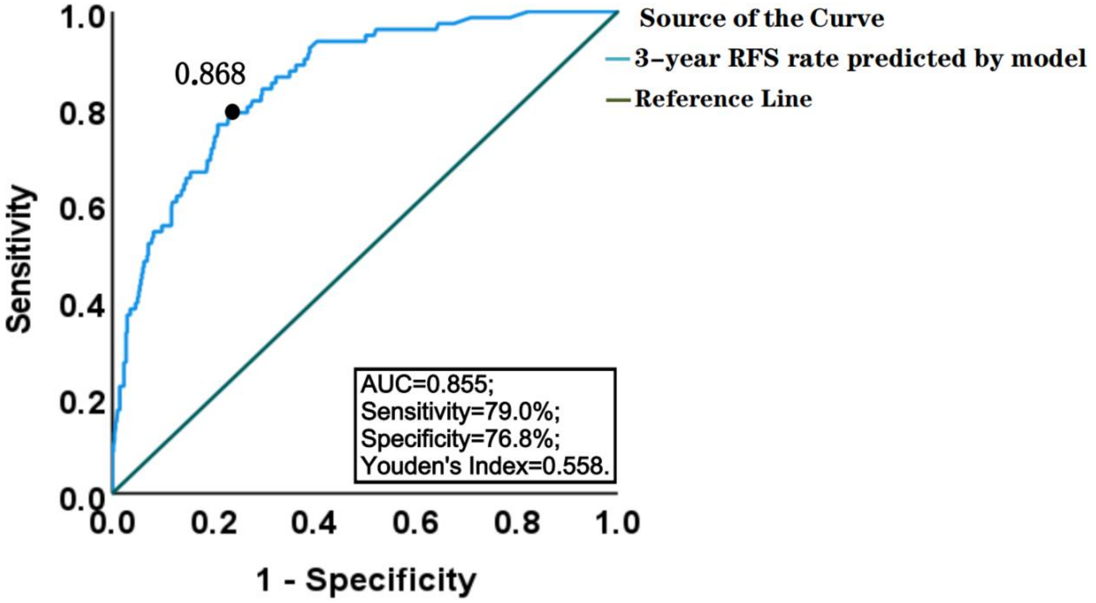

Supplementary Figure 7 The ROC curve of the 3-year RFS rates (predicted by the nomogram model) for predicting the recurrence of endometrial cancer.

Notes: ‘black dot’ represents the area under the curve (AUC) at this point is the largest, which suggests that the optimal threshold of the 3-year RFS rate (risk threshold of the model) for predicting the recurrence of endometrial cancer is 0.868 (AUC= 0.855; sensitivity=79.0%; specificity=76.8%)

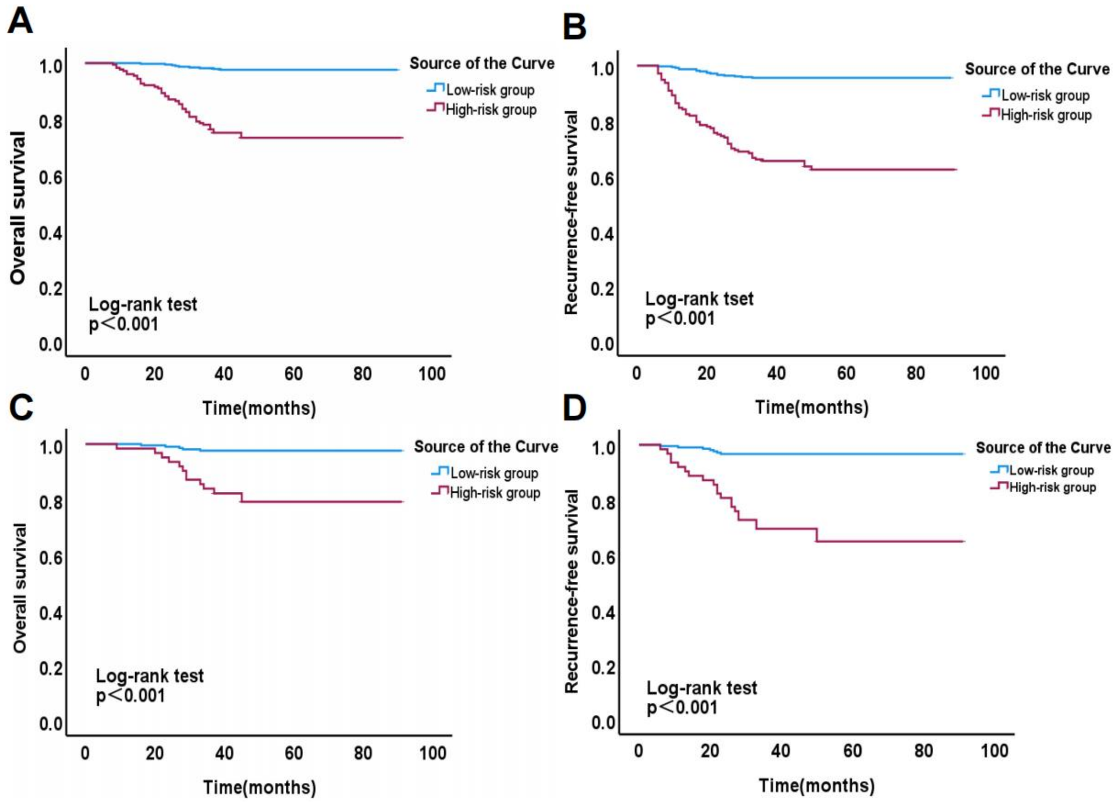

Supplementary Figure 8. Kaplan–Meier survival curve of high-risk and low-risk groups in two cohorts

(A) OS curve and (B) RFS curve of high-risk and low-risk groups in the training cohort; (C) OS curve and (D) RFS curve of high-risk and low-risk groups in the validation cohort.

Supplementary Table 1. Bioinformatics tools.

Databases	Abbreviation	URL
Tumor Immune Estimation Resource	TIMER1.0	https://cistrome.shinyapps.io/timer/
Sangerbox3.0	Sangerbox3.0	http://vip.sangerbox.com/
The University of Alabama Cancer Portal	UALCAN	http://ualcan.path.uab.edu/
Tumor-Immune System Interaction Database	TISIDB	http://cis.hku.hk/TISIDB/
Human Protein Atlas	HPA	https://www.proteinatlas.org/
Kaplan-Meier Plotter	KM Plotter	https://kmplot.com/analysis/
Gene Multiple Association Network Integration Algorithm	GeneMANIA	https://genemania.org/
cBio Cancer Genomics Portal	cBioPortal	https://www.cbioportal.org/
Metascape Gene List Analysis Portal	Metascape	http://metascape.org/
Wei Sheng Xin	WSX	https://www.bioinformatics.com.cn/
Genomics of Drug Sensitivity in Cancer	GDSC	https://www.cancerrxgene.org/

Supplementary Table 2. Correlation of HMGA2 expression with UCEC clinicopathological characteristics.

Characteristics	No. of patients	HMGA2 expression		p value
		Low	High	
**Age(year)**				
＜60	179	85(31.4%)	94(34.7%)	0.411
≥60	363	186(68.6%)	177(65.3%)	
**FIGO Stage**				
Stage I	337	179(66.1%)	158(58.3%)	0.177
Stage II	52	23(8.5%)	29(10.7%)	
Stage III	124	53(19.6%)	71(26.2%)	
Stage IV	29	16(5.9%)	13(4.8%)	
**Grade**				
G1	99	68(25.1%)	31(11.4%)	**＜0.001**
G2	121	72(26.6%)	49(18.1%)	
G3	311	128(47.2%)	183(67.5%)	
High Grade	11	3(1.1%)	8(3.0%)	
**Histological Subtypes**				
EEC	406	214(80.0%)	192(70.8%)	**0.029**
Non-EEC	136	57(21.0%)	79(29.2%)	
**TP53 Expression(n=528)**				
Wild	331	135(51.1%)	196(74.2%)	**＜0.001**
Mutant	197	129(48.9%)	68(25.8%)	
**Therapy**				
No Therapy	239	126(46.5%)	113(41.7%)	0.081
Radiotherapy	106	43(15.9%)	63(23.2%)	
Chemotherapy	70	41(15.1%)	29(10.7%)	
Chemo-radiotherapy	127	61(22.5%)	66(24.4%)	
**Chemotherapy**				
Yes	197	102(37.6%)	95(35.1%)	0.532
No	345	169(62.4%)	176(64.9%)	
**Radiotherapy**				
Yes	233	104(38.4%)	129(47.6%)	**0.03**
No	309	167(61.6%)	142(52.4%)	

Abbreviations: FIGO, International Federation of Gynecology and Obstetrics; EEC, endometrioid carcinoma;

Supplementary Table 3. Correlation of HMGA2 expression with UCEC clinicopathological characteristics in the training cohort.

**Characteristics**	**No. of patients**	**HMGA2 expression**		**p value**
		Low	High	
**Age(year)**				
＜60	418	250(77.2%)	168(71.2%)	0.109
≥60	142	74(22.8%)	68(28.8%)	
**BMI** median(kg/m2)		23.85	24.74	
Mean (± SD)	-	24.37(±3.86)	24.71(±3.71)	0.301
Range	-	16.35-45.72	16.77-41.87	
**FIGO Stage**				
I	381	241(74.4%)	140(59.3%)	
II	62	32(9.9%)	30(12.7%)	**＜0.001**
III	117	51(15.7%)	66(28%)	
**Lymphovascular Space Invasion**				
No	419	257(79.3%)	162(68.6%)	**0.004**
Yes	141	67(20.7%)	74(31.4%)	
**CA125(kU/ml)**				
≤35	415	248(76.5%)	167(70.8%)	0.123
＞35	145	76(23.5%)	69(29.2%)	
**Cervical Stromal Invasion**				
No	481	283(87.3%)	198(83.9%)	0.247
Yes	79	41(12.7%)	38(16.1%)	
**Myometrial Invasion**				
No	411	233(71.9%)	178(75.4%)	0.353
Yes	149	91(28.1%)	58(24.6%)	
**Histological Subtypes**				
G1-G2 EEC	413	251(77.5%)	162(68.6%)	
G3 EEC	58	31(9.6%)	27(11.4%)	**0.048**
Non-EEC	89	42(13.0%)	47(19.9%)	
**P53 Expression**				
Normal	362	218(67.3%)	144(61.0%)	0.126
Abnormal	198	106(32.7%)	92(39.0%)	
**Adjuvant Treatment**				
Follow-up	199	134(41.4%)	65(27.5%)	
Radiotherapy	176	101(31.2%)	75(31.8%)	**0.002**
Chemotherapy	19	11(3.4%)	8(3.4%)	
Chemo-radiotherapy	166	78(24.1%)	88(37.3%)	

Abbreviations: BMI, body mass index; FIGO, International Federation of Gynecology and Obstetrics; EEC, endometrioid carcinoma.

Supplementary Table 4. Clinicopathological characteristics of the training cohort and the validation cohort.

Characteristics	Training Cohort	Validation Cohort	p value
	n=560	n=272	
**Age**(year)			
＜60	418(74.6%)	215(79.0%)	0.163
≥60	142(25.4%)	57(21.0%)	
**BMI** median(kg/m2)			
Mean (± SD)	24.51(±3.79)	24.51(±3.91)	0.996
Range	16.35-45.72	16.35-45.72	
**FIGO Stage**			
I	381(68.0%)	193(71.0%)	
II	62(11.1%)	29(10.7%)	0.661
III	117(20.9%)	50(18.4%)	
**LVSI**			
No	419(74.8%)	207(76.1%)	0.688
Yes	141(25.2%)	65(23.9%)	
**CA125**（kU/ml）			
≤35	415(74.1%)	204(75.0%)	0.782
＞35	145(25.9%)	68(25.0%)	
**Cervical Stromal Invasion**			
No	481(85.9%)	238(87.5%)	0.526
Yes	79(14.1%)	34(12.5%)	
**Myometrial Invasion**			
No	411(73.4%)	195(71.7%)	0.605
Yes	149(26.6%)	77(28.3%)	
**Histological Subtypes**			
G1-G2 EEC	413(73.8%)	212(77.9%)	
G3 EEC	58(10.4%)	31(11.4%)	0.126
Non-EEC	89(15.9%)	29(10.7%)	
**P53 Expression**			
Normal	362(64.6%)	175(64.3%)	0.931
Abnormal	198(35.4%)	97(35.7%)	
**Adjuvant Treatment**			
Follow-up	199(35.5%)	118(43.4%)	
Radiotherapy	176(31.4%)	69(25.4%)	0.135
Chemotherapy	19(3.4%)	10(3.7%)	
Chemo-radiotherapy	166(29.6%)	75(27.6%)	

Abbreviations: BMI, body mass index; FIGO, International Federation of Gynecology and Obstetrics; LVSI, lymphovascular space invasion; EEC, endometrioid carcinoma.

Supplementary Table 5. Univariate and multivariate analysis of factors predicting EC recurrence in the training cohort.

Characteristics	Univariate analysis			Multivariate analysis		
	Hazard ratio	95% CI	P-value	Hazard ratio	95% CI	P-value
**Age (**≥60 vs <60)	2.660	1.717-4.119	**<0.001**	1.885	1.184-3.000	**0.008**
**BMI**	0.963	0.907-1.024	0.230			
**FIGO Stage**						
I	ref		**<0.001**	ref		**0.021**
II	2.705	1.337-5.475	**0.006**	1.266	0.451-3.556	0.655
III	6.801	4.185-11.051	**<0.001**	2.183	1.218-3.915	**0.009**
**LVSI (**Yes vs No)	4.042	2.609-6.262	**<0.001**	2.119	1.311-3.426	**0.002**
**CA125(** > 35 vs ≤35)	2.361	1.521-3.664	**<0.001**	1.653	1.032-2.647	**0.037**
**Cervical Stromal Invasion (**Yes vs No)	2.078	1.254-3.444	**0.005**	1.358	0.648-2.845	0.417
**Myometrial Invasion** (Yes vs No)	3.538	2.286-5.476	**<0.001**	1.930	1.180-3.157	**0.009**
**Histological Subtypes**						
G1-G2 EEC	ref		**<0.001**	ref		**0.030**
G3 EEC	3.537	1.921-6.512	**<0.001**	1.790	0.944-3.396	0.075
Non-EEC	5.553	3.426-9.001	**<0.001**	2.068	1.185-3.609	**0.011**
**P53 Expression** (Abnormal vs Normal)	2.076	1.342-3.210	**0.001**	1.801	1.151-2.817	**0.010**
**Adjuvant Treatment** (Yes vs No)	3.062	1.692-5.543	**<0.001**	0.966	0.469-1.881	0.918
**HMGA2(**High vs Low)	2.010	1.293-3.125	**0.002**	1.603	1.012-2.540	**0.044**

Abbreviations: BMI, body mass index; FIGO, International Federation of Gynecology and Obstetrics; LVSI, lymphovascular space invasion; EEC, endometrioid carcinoma.
